# Senescent Tumoral HLA-E Reshapes Microenvironment through Impairing NK Cell-Dendritic Cell-T Cell Network in Malignant Pleural Effusion from Lung Cancer

**DOI:** 10.7150/ijbs.116499

**Published:** 2025-08-11

**Authors:** Ying-Ming Tsai, Jen-Yu Hung, Yu-Yuan Wu, Hung-Pei Tsai, Kuan-Li Wu, Tai-Huang Lee, Hung-Hsing Chiang, Wei-An Chang, Hsiao-Chen Lee, Sheng-Feng Pan, Kai-Chien Chuang, Shu-Fang Jian, Ling-Yu Wu, Ya-Ling Hsu

**Affiliations:** 1 Division of Pulmonary and Critical Care Medicine, Department of Internal Medicine, Kaohsiung Medical University Hospital, Kaohsiung Medical University, No.100, Tzyou 1 st Road, Kaohsiung 807378, Taiwan.; 2 Division of Pulmonary Medicine, Department of Internal Medicine, Kaohsiung Medical University Gangshan Hospital, Kaohsiung Medical University, No. 8, Jie-An Road, GangShan Dist., Kaohsiung 820, Taiwan.; 3 School of Medicine, College of Medicine, Kaohsiung Medical University, No. 100, Shiquan 1 st Rd., Sanmin Dist., Kaohsiung City 807378, Taiwan.; 4 Graduate Institute of Medicine, College of Medicine, Kaohsiung Medical University, No. 100, Shiquan 1 st Rd., Sanmin Dist., Kaohsiung 807378, Taiwan.; 5 Division of Neurosurgery, Department of Surgery, Kaohsiung Medical University Hospital, No.100, Tzyou 1 st Road, Kaohsiung 807378, Taiwan.; 6 Division of Thoracic Surgery, Department of Surgery, Kaohsiung Medical University Hospital, Kaohsiung Medical University, No.100, Tzyou 1 st Road, Kaohsiung 807378, Taiwan.; 7 Division of Thoracic Surgery, Department of Surgery, Kaohsiung Medical University Gangshan Hospital, Kaohsiung Medical University, No. 8, Jie-An Road, GangShan Dist., Kaohsiung 820, Taiwan.; 8 Department of Respiratory Care, College of Medicine, Kaohsiung Medical University, Kaohsiung 807, Taiwan.; 9 Division of Plastic Surgery, Department of Surgery, Kaohsiung Medical University Hospital, Kaohsiung Medical University, No.100, Tzyou 1 st Road, Kaohsiung 807378, Taiwan.; 10 Division of Plastic Surgery, Department of Surgery, Kaohsiung Municipal Siaogang Hospital, Kaohsiung Medical University, No. 482, Shanming Rd. Siaogang Dist., Kaohsiung 821, Taiwan.; 11 Drug Development and Value Creation Research Center, Kaohsiung Medical University, No. 100, Shiquan 1 st Rd., Sanmin Dist., Kaohsiung 807378, Taiwan.; 12 National Pingtung University of Science and Technology, Department of Biological Science and Technology, No. 1, Shuefu Road, Neipu Township, Pingtung County 912301, Taiwan.

**Keywords:** dendritic cell, HLA-E, nature killer cell, pleural metastasis, senescent cancer cell

## Abstract

**Background:** Malignant pleural effusion (MPE) is ominous in lung cancer patients. However, comprehensive studies of both innate and adaptive immune responses within the pleural tumor microenvironment remain limited.

**Methods:** We collected samples from patients with heart failure and lung cancer-MPE. By single-cell RNA sequencing, we analyzed alternations in cancer cells, NK cells, DCs, and T cells. Key cytokines involving in cell-cell interactions were quantified using Luminex or ELISA, while HLA-E and aging markers were assessed via immunohistochemistry.

**Results:** Our findings revealed that CD56⁺CD16⁺ and CD56⁻CD16⁻ NK cells exhibited reduced cytotoxicity, mainly through HLA-E-expressing senescent cancer cells interacting with NK cells inhibitory receptor, leading to NK cell dysfunction and reduced XCL2 expression, which might impair cDC1 recruitment. Consequently, aDC2 cells evolved into exhausted phenotype, resulting in inadequate T cell activation. In CD8 T cells, transcription factors such as *FOXO1* contributed to diminished cytotoxicity. Despite presence of GZMA CD4 T cells, their cytotoxicity was suppressed in MPE. Th1-like and Th2-like regulatory T cells further inhibited CD4 T cell responses. Key molecules, CXCL16, BAG6, and IL-7, bridging innate and adaptive immunity conferred poor prognosis.

**Conclusions:** Our study demonstrates that senescent cancer cells promote immunoevasion through HLA-E, suppressing NK cell cytotoxicity, impairing DC function, and disrupting T cell activation. Cell-cell interaction and imbalanced Th1/Th2 contribute to microenvironmental remodeling, driving disease progression. These findings provide insights into the immunological landscape and therapeutic targets for intervention.

## Introduction

Lung cancer remains the leading cause of cancer-related deaths worldwide. Despite advancements in diagnostic methods, the majority of lung cancer patients are still diagnosed at late stages, leaving them with limited chances for a cure. It is estimated that approximately 15% of non-small cell lung cancer (NSCLC) patients present with malignant pleural effusion (MPE), which results from the accumulation of exudative fluid in the pleural space containing cancer cells 1, 2 and excessive plasma leakage due to a hyperpermeable pleural vasculature 3. Lung adenocarcinoma (LUAD) is the most common histological type associated with MPE. MPE is typically caused by metastatic diseases, with lung cancer in men and breast cancer in women accounting for 50-65% of cases 4. The excessive accumulation of pleural fluid not only leads to patients' symptoms, such as dyspnea and chest tightness but also significantly shortens the survival of affected patients. In lung cancer, MPE precludes the possibility of curative surgery 5. Despite advancements in cancer treatment, the management of MPE remains primarily palliative, with median survival ranging from 3 to 12 months 6, 7. Therefore, identifying an optimal strategy to prevent its formation or effectively treat this condition is an urgent clinical need.

The metastatic tumor microenvironment in the pleural cavity consists of different tumor cells, immune cells, and stromal cells with their surrounding extracellular matrix 8, 9. MPE is a complex tumor-associated microenvironment rich in immune cells. Compared to blood, immune cells in MPE are enriched with innate immune cells, such as natural killer (NK) cells, macrophages, and dendritic cells (DCs), as well as adaptive immune cells, including T cells and B cells 10. NK cells responding to malignant transformation is regulated through activating and inhibitory receptors, which determine their cytotoxic activity and immune surveillance capability. NK cells release cytokines that influence antigen-presenting cell (APC) infiltration and modulate T cell function 11, 12. Previous studies have shown that NK cells in MPE develop an atypical CD56^bright^CD16⁻ decidual-like phenotype with impaired degranulation capability and reduced perforin release 13. Previous studies have revealed that T cells in MPE express higher inhibitory receptors than those in the peripheral blood. Furthermore, among CD4⁺ and CD8⁺ T cells, T follicular helper cells and the exhausted T cell cluster exhibit the highest levels of inhibitory receptors, respectively 14. However, how innate immune cells and adaptive immune cells, or different types of innate immune cells, collectively influence T cell function in the pleural metastatic niche is not clarified.

Our previous research in lung cancer demonstrated that the immunosuppressive function of lipid-associated tumor-associated macrophages (LA-TAMs) played a crucial role in shaping the metastatic pleural microenvironment, with C5 identified as a predictive marker for survival in epithelial growth factor receptor (EGFR)-mutated lung cancer with MPE 15. In this study, we shifted our focus to the alterations in lymphoid immunity and the interplays between innate immune cells, particularly their impact on T cell function. Furthermore, we explore how pleural metastatic cells underwent senescent phenotype transition and exhibited high HLA-E (human leukocyte antigens-E) expression, driving the pleural immune landscape toward an immunosuppressive niche. Through this research, we presented a comprehensive perspective that extends beyond previous findings, offering a broader and integrative view of the immune dynamics within the MPE microenvironment.

## Materials & Methods

### Patient consent and specimens

All participants were recruited from the Division of Thoracic Surgery and the Division of Pulmonary and Critical Care Medicine at Kaohsiung Medical University Hospital (KMUH) in Kaohsiung, Taiwan. The study was approved by the hospital's Institutional Review Board (KMUH-IRB-20180023; KMUH-IRB-20200038; KMUH-IRB-E(II)-20220175), and all participants provided informed consent. For transcriptome analysis using single-cell RNA sequencing (scRNA-seq), pleural effusion from 4 patients with congestive heart failure as HP and 5 patients with malignant pleural effusion (MPE) caused by lung adenocarcinoma as LCP were analyzed. Additionally, 14 patients with congestive heart failure were included as controls, while 79 patients with lung adenocarcinoma-related MPE were examined for the analysis of associated secreted factors.

### scRNA-seq and analysis

Single-cell barcoding of thawed samples and complementary DNA (cDNA) library preparation were carried out following the manufacturer's instructions using the Single Cell 3′ Reagent Kits v3 (10x Genomics, USA). The scRNA-seq protocol and criteria followed the criteria and settings from our previous publication in the *Theranostics* 15.

### Partition-based graph abstraction (PAGA)

Partition-based graph abstraction (PAGA) was used to compute the relationships between cDC, activated DC, and transitional DC using Scanpy (v1.10.2) 16, 17. The analysis followed the Scanpy tutorial with some custom modifications. First, DC subclusters were normalized and genes expressed in fewer than three cells were excluded. The top 2000 highly variable genes (HVGs) were selected in Scanpy using Seurat v3 method 18. Next, a Diffusion Map (diffmap) with a neighborhood graph (n_neighbors = 10) was constructed and a Force-directed graph was generated using ForceAtlas2 19. All of which were performed within Scanpy. The Louvain algorithm 20 was applied to identify clusters (resolution = 1.0) in Scanpy. Finally, the 18^th^ Louvain cluster was selected as the root cell, and the gene expression paths were figured using Diffusion Pseudotime (DPT) in Scanpy 16, 21.

### Trajectory analysis by monocle

The trajectory of cell transitions and key regulatory genes were analyzed using Monocle 2 (v2.26.0). Genes with a significant q-value (< 0.05) from the autocorrelation analysis were selected. These identified genes were subsequently grouped into distinct modules using Uniform Manifold Approximation and Projection (UMAP).

### Differential expression analysis

Differential expression was conducted to investigate key molecular signatures. Signature genes were defined as those expressed in more than 50% of cells in at least one of the two subsets or HP/LCP, with a differential expression change greater than twofold (log_2_ FC > 1). Pathway enrichment analysis was performed to explore enriched biological processes within clusters, utilizing the DAVID platform (https://david.ncifcrf.gov/) and Ingenuity Pathway Analysis (IPA). These analyses aimed to identify potential molecular functions associated with the identified gene modules. Additionally, the survival impact of specific gene sets was assessed using the Gene Set Cancer Analysis (GSCA) platform (http://bioinfo.life.hust.edu.cn/GSCA/#/). Gene Set Variation Analysis (GSVA), an unsupervised algorithm, was executed to calculate enrichment scores of specific gene sets (Molecular Signatures Database (MSigDB), http://www.gsea-msigdb.org/gsea/msigdb/collections.jsp).

### InferCNV

To generate a new gene-cell matrix, primary cancer cells and pleural cancer cells were lumped together. The somatic large-scale chromosomal copy number variation (CNV) score of cancer cells was determined using the InferCNV R package (v1.6.0). Data preparation involved creating a raw counts matrix, an annotation file, and a gene/chromosome position file, following the guidelines provided by the InferCNV repository (https://github.com/broadinstitute/inferCNV). For reference cell selection, mesothelial cell in HP was used as controls for pleural metastatic cancer cells. Default settings were applied, with a cutoff value of 0 and a denoising threshold of 0.2.

### Cell-cell interactions

To explore cell-cell interactions among various cell types—including *HLA-E* positive cancer cells, *HLA-E* negative cancer cells, dendritic cells (DCs), lipid-associated tumor-associated macrophages (LA-TAMs), CD8 T cell subsets, CD4 T cell subsets, and NK cell subsets—the CellChat package and the CelltalkDB database (http://tcm.zju.edu.cn/celltalkdb/) 22 were utilized. These tools were employed to assess the significance of ligand-receptor interactions across different cell clusters. Ligand-receptor pairs with a p-value of less than 0.05 were considered to represent significant interactions between cell types.

### The analysis of various cytokine/chemokines in pleural effusion

The levels of various soluble factors in the pleural effusion were measured using enzyme-linked immunosorbent assay (ELISA) kits for human BAG6 (FineTest, Cat. EH14786). Other factors, including Interferon-γ, TNF-α, IL-12, IL-4, IL-5, IL-10, IL-7, IL-16, CCL22, CX3CL1, CXCL16, IL-6, IL8, Granzyme A and RANTES were assessed using a Luminex assay (R&D Systems).

### Flow cytometry

Pleural effusion samples from lung cancer patients were centrifuged at 1500 rpm for 15 minutes at 4°C to collect the cellular fraction. The cell pellets were resuspended in phosphate-buffered saline (PBS) and stained with the following fluorochrome-conjugated antibodies: anti-CD8 (PE-Cy7, cat. No. 557746, BD Pharmingen), anti-CD4 (FITC, cat. No. 555346, BD Pharmingen), anti-GZMA (PE, cat. No. 51-68395X, BD Pharmingen), and anti-CD45 (APC, cat. No. 555485, BD Pharmingen). Appropriate isotype controls were included to assess nonspecific binding. For intracellular staining of GZMA, cells were fixed and permeabilized using a fixation/permeabilization kit (according to the manufacturer's instructions). Flow cytometric analysis was performed using a BD FACSLyric™ flow cytometer (BD Biosciences, San Jose, CA, USA). Data were analyzed by gating on CD45⁺CD8⁻ cells, followed by identification of the GZMA CD4 population within this gate.

### Immunohistochemistry (IHC)

p21 and HLA-E expression were determined by IHC in pleural metastatic cancer from 5 samples. Clinical formalin-fixed paraffin-embedded (FFPE) specimens were obtained from the KMUH. For each specimen, a pathologist reviewed the hematoxylin and eosin stain slides. Paraffin sections were dewaxed and processed for antigen retrieval (cat. No. C9999, Sigma-Aldrich). Three μm sections were treated with H_2_O_2_ for 10 min to quench endogenous peroxidase, pre-incubated in Protein Block (TA-060-PBQ, Thermo Fisher) solution 10 min and then exposed to anti-p21 (cat. No. ZRB1141, Merck; 1:1,000), and anti-HLA-E (cat. No. ab2216, Abcam, 1:100) antibodies for 60 min and then primary antibody amplifier Quanto (TL-060-QPB, Thermo Fisher) 10 min. After washing, the sections were incubated with HRP Polymer Quanto (TL-060-QPH) for 10 min, developed with 3,3'-Diaminobenzidine (DAB) (TA-060-QHSX and TA-002-QHCX) for 5 min and then co-stained with counterstain with hematoxylin (MHS16, Sigma-Aldrich) for 10 min, dehydrated, cleared in xylene and coverslipped.

### Statistical analysis

All statistical analyses were conducted using GraphPad Prism software (version 9.0.0). Data were expressed as mean ± standard deviation (SD). For comparisons between two groups, an unpaired Student's t-test was applied, while one-way analysis of variance (ANOVA) was used for comparisons involving multiple groups. A two-tailed p-value of less than 0.05 was considered statistically significant. Kaplan-Meier survival analysis and the log-rank test were used to assess differences in overall survival between patient groups stratified by various factor levels in the pleural fluid.

## Results

### The *HLA-E-KLRD1* interaction acts as a mechanism by which cancer cells suppress NK cell activation

We expanded our previously established MPE single-cell RNA-seq database as the framework for our study (HP = 4, LCP = 5) (sTable 1). We then performed clustering analysis on myeloid cell lineages, including monocytes, dendritic cells (DCs), macrophages/monocyte and neutrophils, as well as lymphoid lineages, including CD4 T cells, CD8 T cells, CD4CD8 double-negative (DN) CD3E^+^ cells, CD4CD8 double-positive (DP) CD3E^+^ cells, B cells and NK cells (sFigure 1A,B). The results showed that macrophages and T cells were the predominant immune cells in the pleural effusion, regardless of the pleural effusion of HP or LCP (sFigure 1C). Among them, most immune cells, except plasma cells, were significantly increased in MPE of LCP (sFigure 1D).

**Figure 1:**
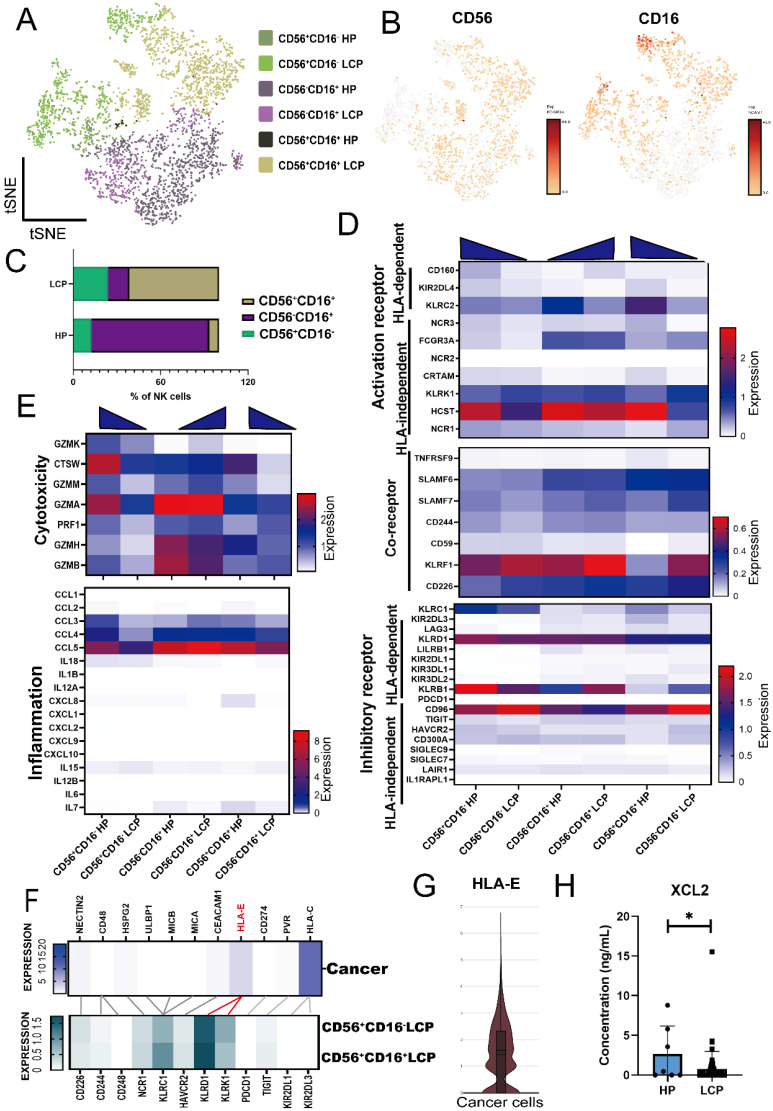
** Landscape of NK cell subsets in MPE.** (A) t-distributed stochastic neighbor embedding (tSNE) projection showing three clusters of NK cells in HP (n = 4) and LCP (n = 5). (B) Gene expression of NK cell surface markers: CD56 (*NCAM1*) and CD16 (*FCGR3A*). (C) Comparison of ratio in total cells between HP and LCP. (D) Heatmap of activating and inhibitory receptor expressions in NK subsets. (E) Cytotoxic and inflammatory profiles across three subsets of NK cells. (F) Tumor antigen-receptor interactions between cancer cells and two major subsets of NK cells in MPE. (G) Violin plot of HLA-E expression in cancer cells. (H) Quantification of XCL2 expression in pleural fluid was assessed by ELISA, comparing HP with LCP samples. * *p* < 0.05. HP: pleural effusion of heart failure; LCP: pleural effusion of lung cancer.

Our previous research has identified the role of mesothelial cells and myeloid cells in MPE; therefore, we focused on lymphoid lineages in this study. We reclustered the NK cell population and classified them into three major subsets based on CD56 and CD16 expression: CD56^+^CD16^+^, CD56^+^CD16^-^, and CD56^-^CD16^+^ NK cells (Figure 1A and 1B). In the pleural effusion of HP, CD56^-^CD16^+^ NK cells were the predominant subset, whereas in MPE of LCP, CD56^+^CD16^+^ NK cells were the major population. Additionally, CD56^+^CD16^-^ NK cells were more abundant in MPE of LCP compared to pleural effusion of HP (Figure 1C). We analyzed the expression of activation/inhibition-related genes in NK cells. And we found that activating receptors such as *HCST* and *KLRC2* were significantly downregulated in CD56^+^CD16^+^ and CD56^+^CD16^-^ NK cells from MPE of LCP, whereas the inhibitory receptor *CD96* was notably upregulated compared to HP (Figure 1D).

Additionally, genes associated with NK cell cytotoxicity, including *CTSW*, *GZMA*, and *GZMB*, as well as pro-inflammatory cytokines, *CCL4* and *CCL5*, were downregulated in LCP (Figure 1E). Analysis of 11 antigens that regulated NK cell activity in pleural cancer revealed that *HLA-E* and *HLA-C* were the predominant tumor-associated antigens. However, while the corresponding receptor of *HLA-C* was expressed at low levels in CD56^+^CD16^-^ and CD56^+^CD16^+^ NK cells in MPE, the inhibitory receptor *KLRD1*, which corresponds to its ligand, *HLA-E*, was highly expressed (Figure 1F and 1G). These findings suggest that the *HLA-E-KLRD1* axis plays a critical role in suppressing the anti-cancer activity of NK cells in MPE.

We analyzed the differentially expressed genes (DEGs) of CD56^+^CD16^+^ NK cells and CD56^+^CD16^-^ NK cells in pleural effusion of HP and LCP and identified 14 genes downregulated in NK cells from MPE of LCP. These included *CTSW* and *PFN1*, which are associated with NK cell cytotoxicity (sFigure 2A and 2B). Notably, the expression of *XCL2*, a key chemokine involved in dendritic cell (DC) recruitment, was significantly reduced. ELISA results confirmed that XCL2 concentration was significantly lower in MPE from LCP than in pleural effusion from HP (Figure 1H). However, XCL2 levels in the pleural effusion were not associated with overall survival of lung cancer patients with MPE, regardless of EGFR mutation status (sFigure 2C to 2E). Together, these findings suggest that NK cell cytotoxicity and its ability to recruit DCs are significantly suppressed in MPE.

### Senescent cancer cells exhibit high *HLA-E* expression

**Figure 2:**
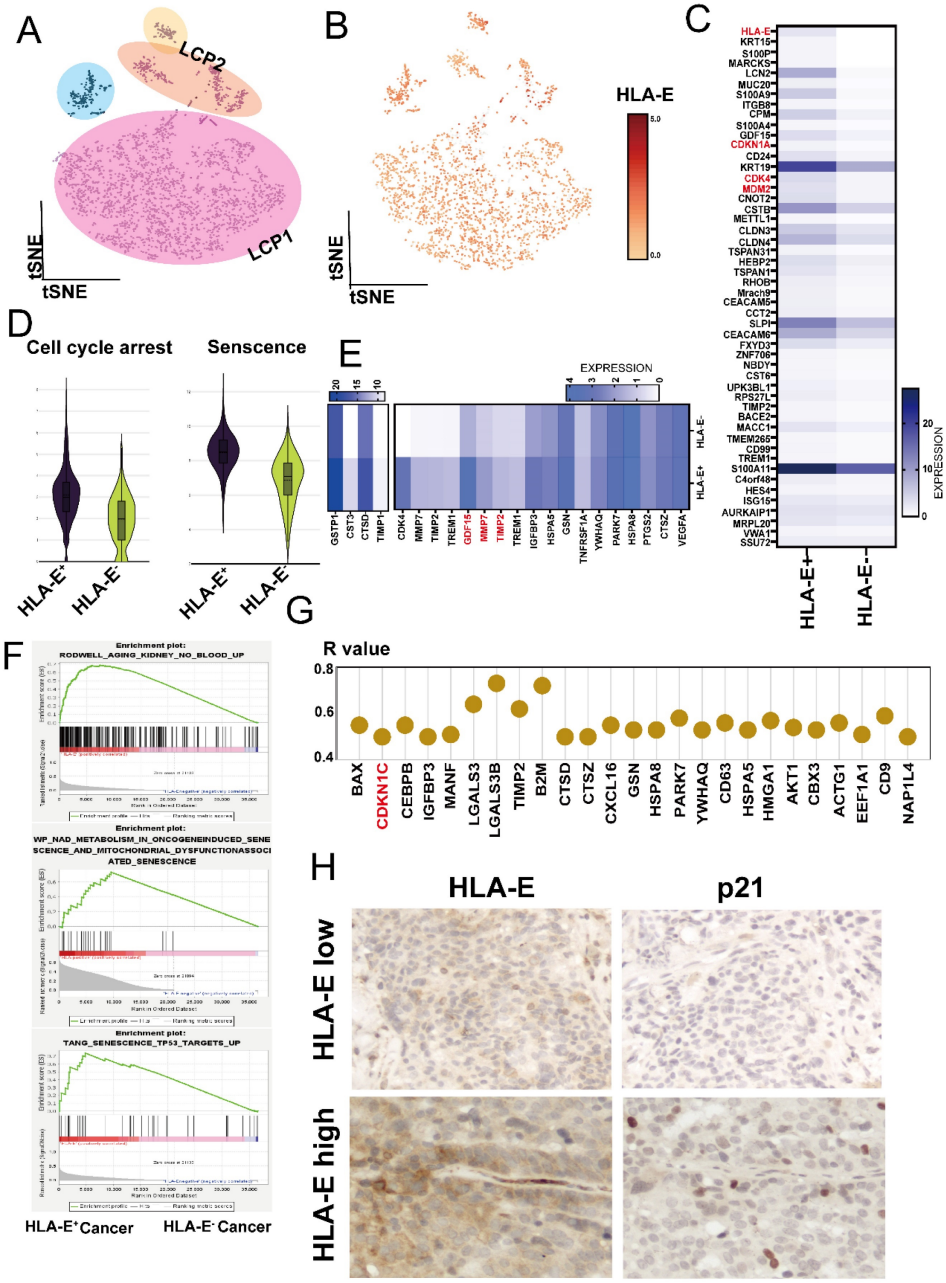
** Deconvolution of *HLA-E* positive cancer cells in LCP. (A)** t-SNE plot presenting cancer cells from MPE of lung cancer patients. **(B)** Differential expression of *HLA-E* in pleural metastatic cancer cells. **(C)** Heatmap comparing distinct gene expression profiles between *HLA-E* positive and *HLA-E* negative cancer cells. **(D)** Cell cycle arrest and senescence-weighted scores in the *HLA-E* positive and *HLA-E* negative cancer cells. **(E)** Heatmap illustrating senescence-associated secretory phenotype gene expression in *HLA-E* positive and *HLA-E* negative cancer cells. **(F)** Enrichment plots reveal the senescence phenotype associated with the *HLA-E* positive cluster. **(G)** The expression of senescence-related genes positively correlated with *HLA-E* within the *HLA-E* positive subset. **(H)** Immunohistochemical analysis of p21 and HLA-E expression in pleural metastatic cancer lesions. Representative images indicate the localization and relative expression levels of p21 and HLA-E in tumor cells within the pleura.

We further investigated the underlying mechanism for HLA-E expression in tumor cells. Using InferCNV analysis, we confirmed that EPCAM^+^ cells represented pleural metastatic cancer cells (Figure 2A and sFigure 3A). Notably, most of the cancer cells expressed *HLA-E* (Figure 2B). When comparing *HLA-E* positive with *HLA-E* negative cancer cells, analysis of the top 50 DEGs revealed several cell cycle-related genes that were highly expressed in *HLA-E* positive cancer cells, including *CDKN1A*, *CDK4*, and *MDM2* (Figure 2C). Using the cell cycle arrest and senescence gene sets extracted from the Cellular Senescence Network (SenNet, http://sennetconsortium.org/), we found that both the cell cycle arrest and senescence scores were higher in *HLA-E* positive cancer cells compared to *HLA-E* negative cancer cells (Figure 2D). Additionally, several soluble factors associated with the senescence phenotype were highly expressed in* HLA-E* positive cancer cells, particularly *GDF15*, *MMP2*, and *MMP7* (Figure 2E). Gene Set Enrichment Analysis (GSEA) further supported that *HLA-E* positive cancer cells exhibited a senescence phenotype (Figure 2F). Correlation analysis showed a strong association between *CDKN1C*, a key senescence marker, and *HLA-E* expression in *HLA-E* positive cancer cells (R > 0.5, p < 0.05) (Figure 2G). Moreover, IHC staining confirmed that HLA-E and p21 were co-expressed at high levels in pleural metastatic cancer (Figure 2H). The ELISA results showed that GDF-15 levels in the MPE of LCP were slightly higher than in HP, although the difference was not statistically significant. However, overall survival data indicated that a high concentration of GDF-15 in MPE was associated with poorer outcomes in lung cancer patients (sFigure 3B and 3C). These findings suggest a strong correlation between senescent phenotype and HLA-E expression in cancer cells.

### Reduced cDC1 and exhausted aDC2 in the pleural microenvironment of lung cancer patients with MPE

**Figure 3:**
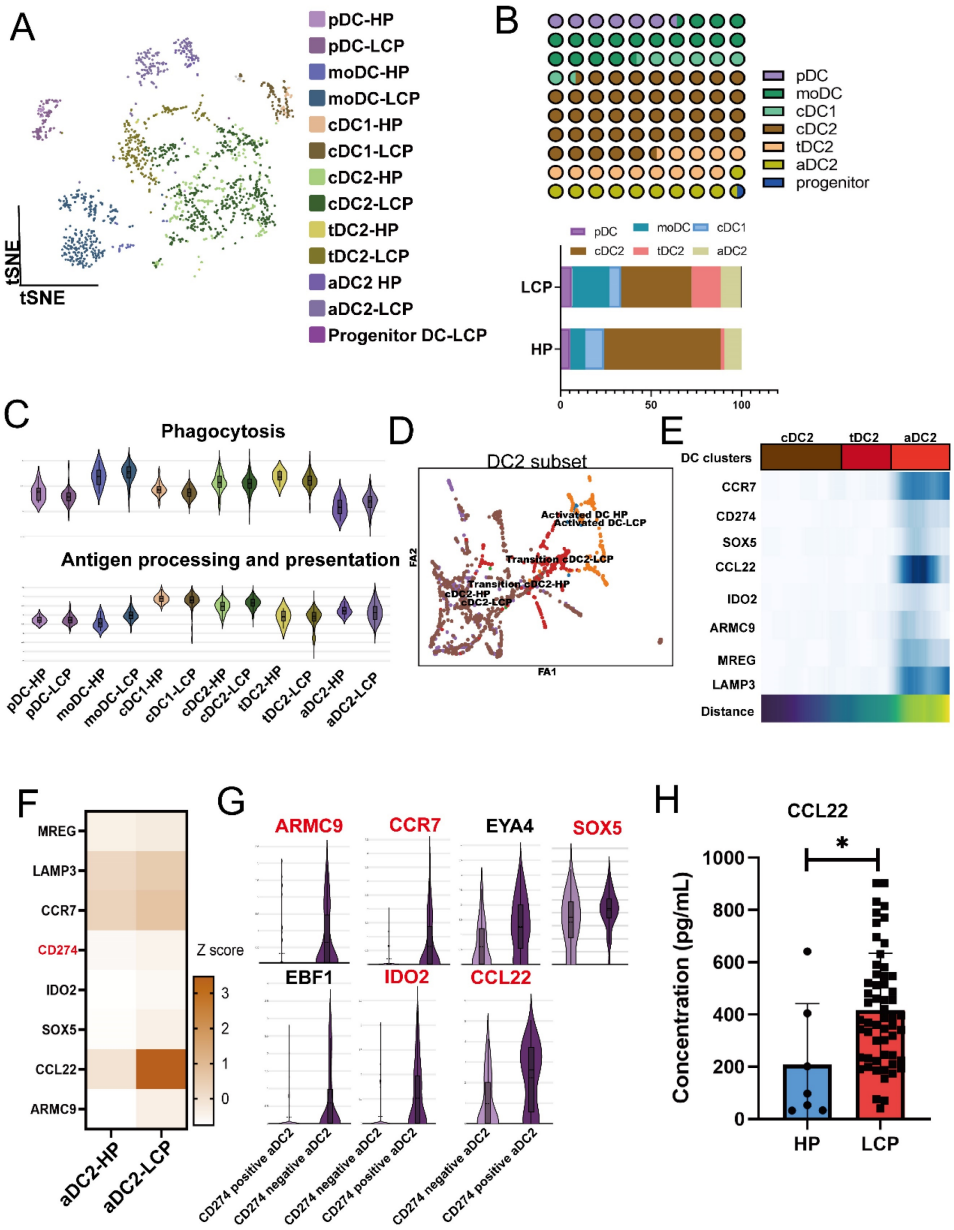
** Distinct phenotypes of aDC2 within MPE. (A)** tSNE map demonstrating 7 subsets of DCs. **(B)** Proportional distribution of 7 DC clusters in HP and LCP. **(C)** Violin plots illustrated functional differences (phagocytosis, antigen processing and presentation) in each DC subtype. **(D)** Partition-based graph abstraction (PAGA) analysis illustrating the developmental transitions among DC subsets. Connectivity strength between clusters reflects transcriptional similarity and suggests possible lineage relationships or maturation pathways across DC states. **(E)** PAGA paths including upregulated genes were involved in the aDC2 transition. **(F)** Heatmap showing DEGs in aDC2 subset from pleural effusion of HP and LCP. **(G)** Violin plots depicting upregulated genes in CD274 negative and CD274 positive aDC2. **(H)** Quantification of CCL22 levels in pleural effusion samples from HP and lung cancer patients. CCL22 levels in pleural effusion were measured by ELISA. * *p* < 0.05.

Since NK cells in MPE exhibited lower XCL2 expression, we further investigated whether DCs in MPE were affected. We reclustered the DC population and classified them into seven subtypes based on cell-specific markers, including pDC, moDC (monocyte-derived DCs), cDC1 (*XCR1*), cDC2 (*CLEC10A*, *CD1C, FCER1A*), activated DC2 (aDC2) *(CCR7*, *LAMP3*), transitional DC2 (tDC2) (*CLEC10A, CD1C, SPRPA*), and progenitor DC (Figure 3A). Among these DC subpopulations, cDC2 was the most abundant. However, compared to the pleural effusion of HP, the proportions of cDC1 and cDC2 were significantly reduced in MPE, whereas aDC2 and tDC2 populations were increased (Figure 3B). Using GO gene set analysis, we examined the phagocytosis and antigen-presenting cell (APC) functions of DC subpopulations. Compared to DCs in HP, cDC2 exhibited higher APC activity but lower phagocytic capacity, whereas aDC2 showed the opposite trend, with lower APC activity but higher phagocytic capacity (Figure 3C). PAGA analysis of cDC2, aDC2, and tDC2 transitions confirmed that cDC2 progressively differentiated into tDC2 before transitioning into aDC2 (Figure 3D). *CCR7*,* CD274*, *SOX5*, *IDO2*, *CCL22*, *ARMC9*, *MRGE*, and *LAMP3* were identified as key genes associated with the transition of aDC2 from cDC2 (Figure 3E and sFigure 4A). Trajectory analysis by Monocle also showed that aDC2 was a terminally differentiated DC, with* CD274, ARMC9, CCL22, IDO2, CCR7,* and* SOX5* involved in the transition (sFigure 4B). Comparison of pleural effusion between HP and LCP patients revealed that these genes were also highly expressed in aDC2 within the MPE of LCP (Figure 3F). Notably, aDC2 exhibited high expression of the exhaustion marker, CD274. When comparing CD274-positive aDC2 and CD274-negative aDC2, we found that *ARMC9, CCR7, SOX5, IDO2,* and* CCL22* were significantly upregulated in CD274-positive aDC2 (Figure 3G). Additionally, these cells showed high expression of CCL22. ELISA analysis further confirmed that concentrations of CCL22 were significantly higher in MPE of LCP compared to pleural effusion of HP (Figure 3H). However, CCL22 levels were not associated with overall survival, regardless of EGFR mutation or wild-type status (sFigure 4CE). These findings suggest that aDC2 exhibits an exhausted phenotype and secretes high levels of CCL22 in the pleural metastatic niche.

### MPE-enriched CXCR3^lo^ CD8 T cells exhibit reduced cytotoxicity due to *FOXO1* upregulation

**Figure 4:**
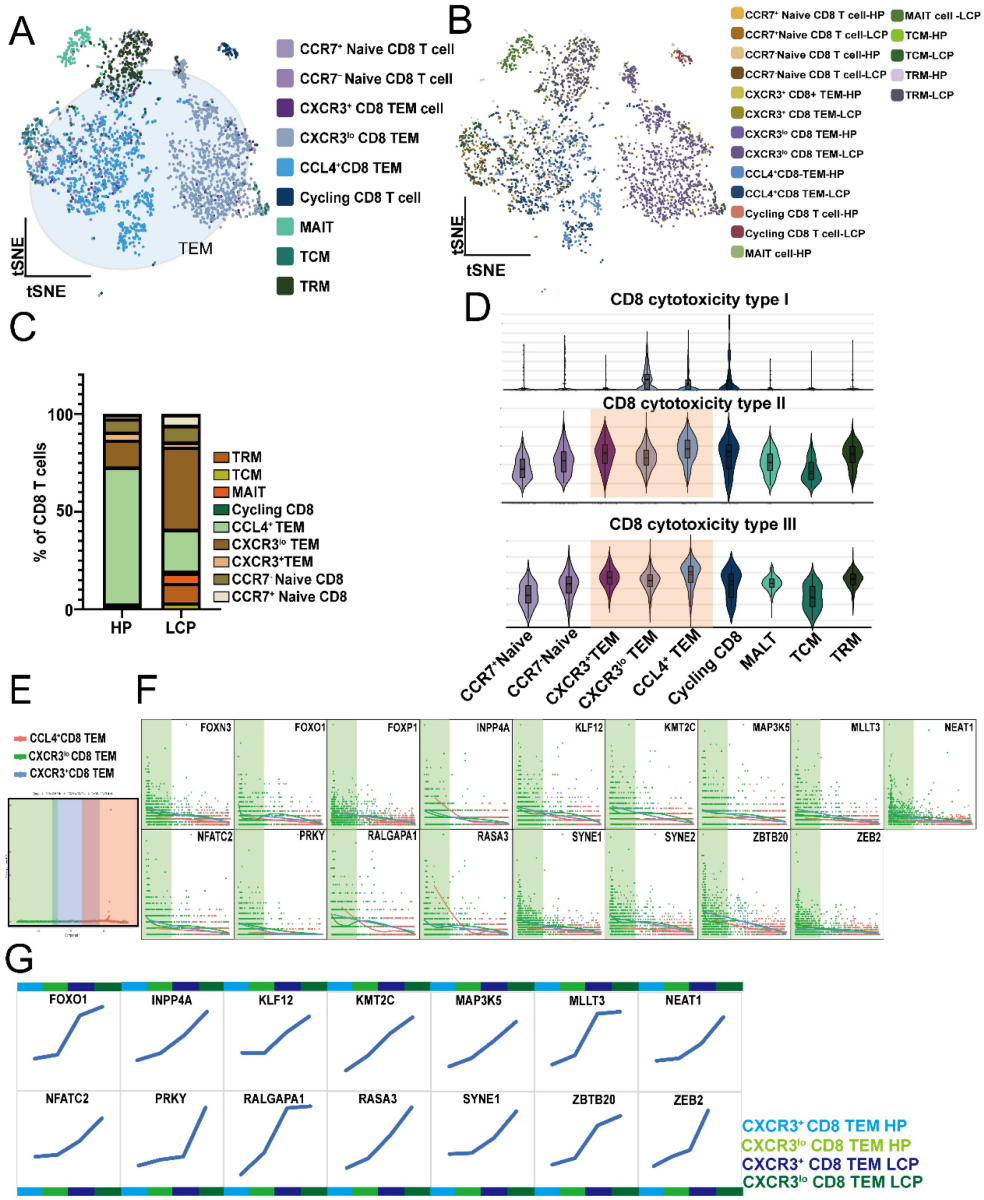
** Profile of dysfunctional CD8 T cells. (A)** tSNE plot illustrating the distribution of 9 transcriptionally distinct CD8 T cell subsets identified from pleural effusion of HP and lung cancer patients. **(B)** Different subsets of CD8 T cells in the pleural effusion of HP and LCP. **(C)** The proportion of various subsets of CD8 T cells in pleural effusion of HP and LCP. **(D)** Violin plots showing the cytotoxic scores among CD8 T cell subsets. Scores were calculated based on the expression of cytotoxic genes (*GZMA, GZMB, GZMH, GZMK PRF1, GNLY etc*). **(E)** Pseudotime trajectory analysis of three effector memory CD8 T cell (CD8 TEM) subsets. **(F)** key genes contributing to the pseudotemporal transition of CD8⁺ TEM subsets. **(G)** Genes of step-up trend during the transition from CXCR3^+^ to CCL4^+^ CD8 TEM.

Due to the exhausted phenotype observed in APC cells, specifically aDCs, we analyzed CD8 T cells. We defined CD8 T cells as CD3E⁺CD8⁺ cells and further clustered them into 6 subpopulations based on specific markers: Naïve CD8 T cells (*SELL*), TEM (effector memory) (*GZMA, GZMB, GZMK, SELL*), Cycling CD8 T cells (*MKI67*), MAIT (mucosal-associated invariant T cells) (*SLC4A10*)*,* TCM (central memory) (*CCR7, SELL, CD27*), and TRM (tissue-resident memory) (*ITGA1*), (Figure 4A). TEM can be divided into CXCR3⁺, CXCR3^lo^, and CCL4⁺ subpopulations. Comparing the percentage of CD8 subpopulations in pleural effusion between HP and LCP patients, CXCR3^lo^ TEM was significantly increased, whereas CCL4⁺ TEM was notably decreased (Figure 4C). Using GO gene sets to analyze the cytotoxicity of various CD8 subpopulations, CCL4⁺ TEM exhibited the highest type II and III cytotoxicity. At the same time, CXCR3^lo^ TEM showed the lowest activity in type II and III cytotoxicity among the three TEM subpopulations (Figure 4D). Trajectory analysis showed that CXCR3^lo^ TEM represented the initial TEM subpopulation, followed by CXCR3⁺ TEM in the intermediate stage, and CCL4⁺ TEM as the terminal subpopulation (Figure 4E). The downregulation of 17 genes was associated with the transition of TEM subpopulations from CXCR3^lo^ TEM to CCL4⁺ TEM (Figure 4F). Comparing the DEGs between TEM in HP and LCP patients, we found that 14 out of these 17 genes (*FOXN3, FOXO1, FOXP1, INPP4A, KLF12, KMT2C, MAP3K5, MLLT3, NEAT1, NFATC2, PRKY, RALGAPA1, RASA3, SYNE2, SYNE2, ZBTB20,* and* ZEB2*) followed the transition from CXCR3^lo^ TEM to CXCR3⁺ TEM and finally to CCL4⁺ TEM. Moreover, at each stage of the TEM transition, 14 genes (*FOXO1, INPP4A, KLF12, KMT2C, MAP3K5, MLLT3, NEAT1, NFATC2, PRKY, RALGAPA1, RASA3, SYNE1, SYNE2, ZBTB20,* and* ZEB2*) exhibited consistently higher expression levels in the TEM subpopulations of LCP patients compared to those in HP patients (Figure 4G). These results suggest that these factors may be involved in the dysfunction of CXCR3^lo^CD8⁺ TEM cells in the MPE of LCP.

### CD4 T cells expressing GZMA play a cytotoxicity phenotype in the pleural microenvironment

**Figure 5:**
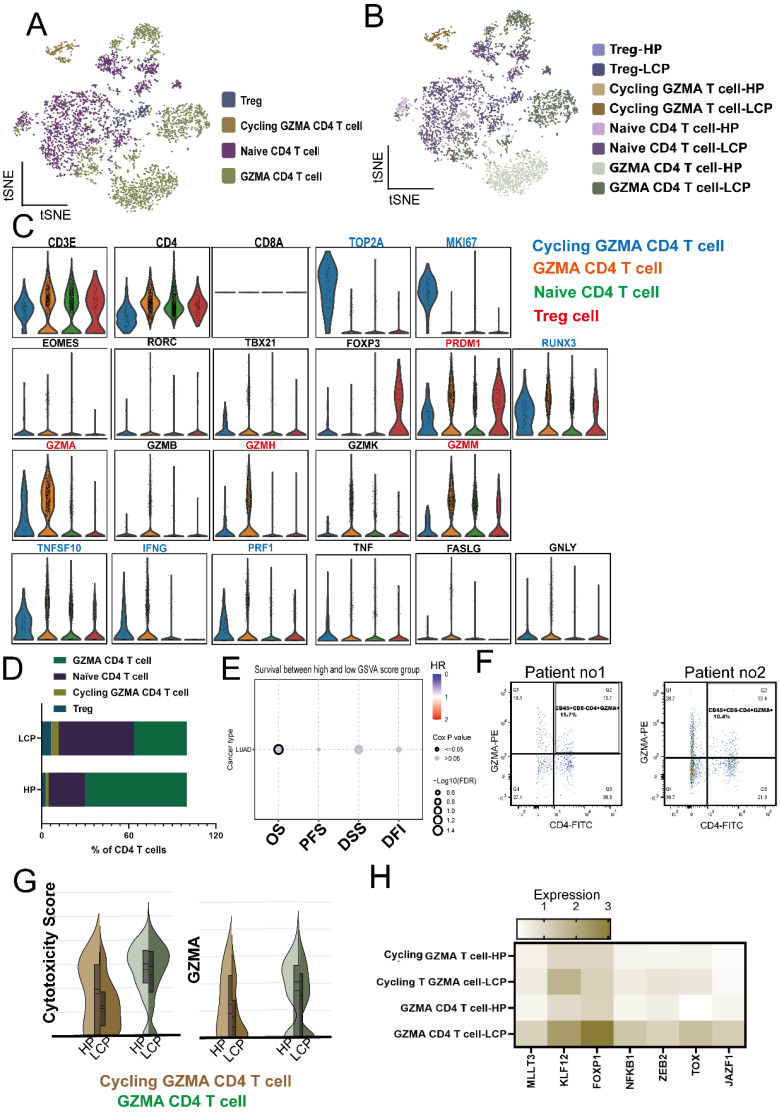
** Functional impairment of cytotoxic CD4 T cells. (A)** Overview of 4 CD4 T cell clusters presented as a tSNE plot. **(B)** Localization of four CD4 T cell subsets in the pleural effusion of HP and LCP. **(C)** Violin plots showing the expression of various genes regulating the cytotoxicity ability of different subsets of CD4 T cells. (D) Bar plots comparing the percentage of CD4 T subsets of HP and LCP. **(E)** GSCA analysis predicting the overall survival rate of lung cancer patients based on the top 100 genes of GZMA CD4 T cells. **(F)** Representative flow cytometry plots show the gating strategy and the presence of CD45⁺CD8⁻CD4⁺GZMA⁺ T cells in MPE from lung cancer patients. These cells were identified based on surface markers CD45, CD4, and CD8, along with intracellular expression of granzyme A (GZMA). **(G)** Violin plots representing cytotoxicity scores and GZMA expression in Cycling GZMA CD4 and GZMA CD4 T cells in the pleural effusion of HP and LCP. **(H)** Heatmap depicting upregulated transcription factors in Cycling GZMA CD4 and GZMA CD4 T cells in the MPE of LCP.

Next, we analyzed CD4 T cells, which are defined as CD3E⁺CD4⁺CD8⁻ cells. Based on specific markers, we classified them into regulatory T cells as Tregs (*FOXP3*), Naïve CD4 T cells (*CCR7, SELL*), and two distinct subsets of CD4 T cells: GZMA CD4 T cells, which express *GZMA* but lack *MKI67*, and Cycling GZMA CD4 T cells, which co-expressing *GZMA* and *MKI67* (Figure 5A and 5B). The lytic granule genes identified in the scRNA-seq analysis were exclusively upregulated in the clusters of GZMA and Cycling GZMA CD4 T cells (Figure 5C). Notably, the GZMA CD4 T cell subset exhibited elevated expression of genes associated with effector and cytolytic functions in CD4 T cells, including *GZMA*, *GZMH*, *GZMM*, and *PRDM1*, while also showing moderate expression of anti-cancer-related genes such as *IFNG*, *PRF1*, and *TNFSF10*. In contrast, Cycling GZMA CD4 T cells expressed *GZMA* and demonstrated a marked upregulation of anti-cancer-related genes, including *IFNG*, *PRF1*, and *TNFSF10* (Figure 5C). Compared to pleural effusion in HP, the proportion of Cycling GZMA CD4 T cells significantly increased in MPE of LCP, whereas the proportion of GZMA CD4 T cells notably decreased (Figure 5D). The GSVA assay revealed that the top 100 gene set highly expressed in GZMA CD4 T cells was associated with better overall survival in LUAD patients, suggesting that the presence of GZMA CD4 T cells may provide potential benefits for lung cancer patients (Figure 5E, sTable 2). Flow cytometry analysis also revealed the presence of CD45⁺CD8⁻CD4⁺GZMA⁺ cells in the MPE of lung cancer patients (Figure 5F). By comparing Cycling GZMA CD4 and GZMA CD4 T cells in the pleural effusion between HP and LCP, we found that both the cytotoxicity score and *GZMA* expression were significantly reduced in the MPE of LCP (Figure 5G). Analysis of DEGs in Cycling GZMA and GZMA CD4 T cells from pleural effusion of HP and LCP revealed that seven transcription factors (*MLLT3, KLF12, FOXP1, NFKB1, ZEB2, TOX, JAZF1*) were upregulated in both cycling GZMA CD4 and GZMA CD4 cells in MPE of LCP (Figure 5H). The results suggested that CD4 T cells expressing GZMA are of ability to anti-cancer.

### Tregs presenting in the pleural effusion exhibit Th1 and Th2-like phenotypic profiles, and simultaneously express both *CTLA4* and *TIGIT*

**Figure 6:**
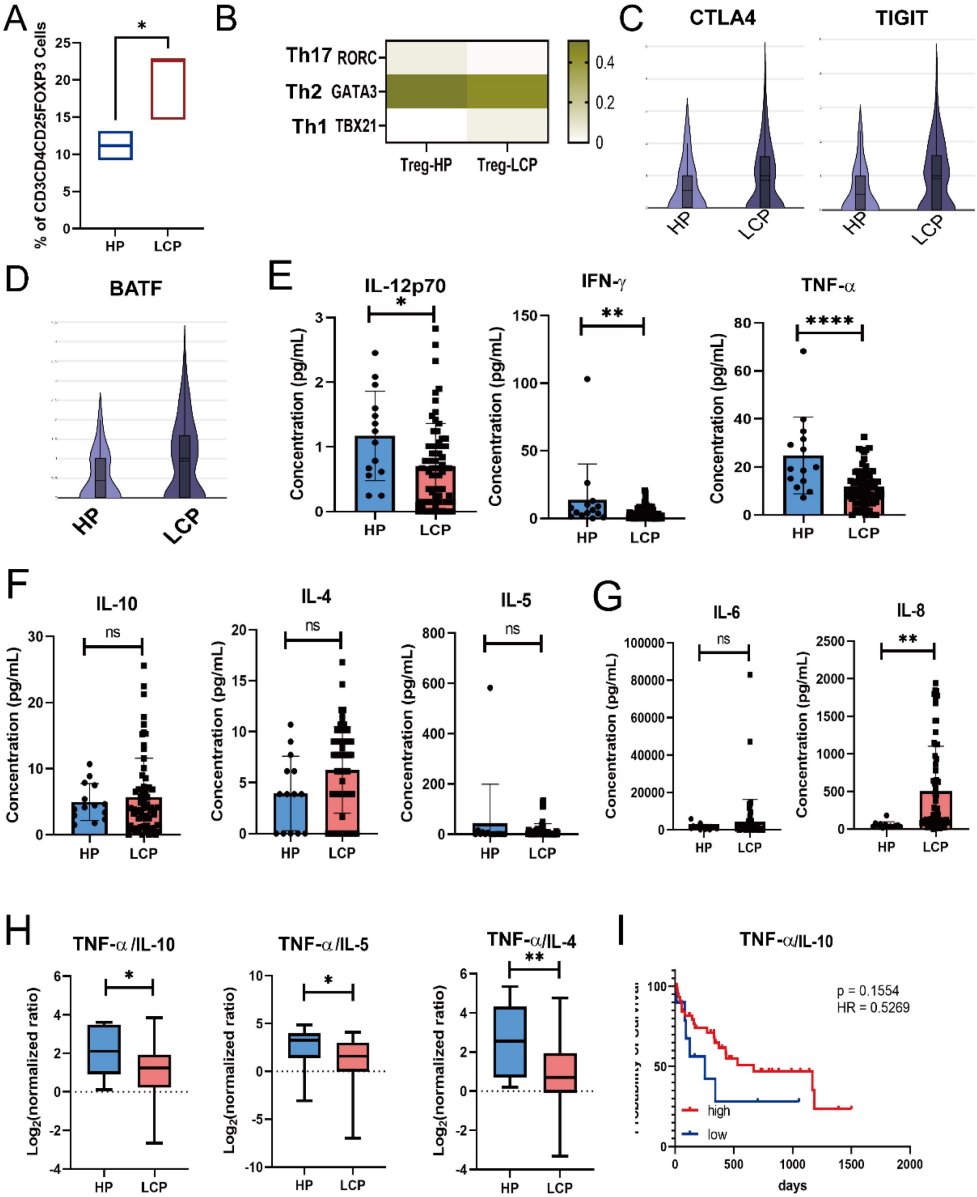
** Tregs were associated with poor survival in LCP patients. (A)** Box plot showing the percentage of Treg (CD4^+^CD25^+^FOXP3^+^) in the pleural effusion of HP and LCP by flow cytometry. **(B)** Heatmap illustrating the expression of helper T-specific transcription factors in Tregs. **(C)** Violin plots depicting *CTLA4* and *TIGIT* expression levels in Treg in the pleural effusion of HP and LCP. **(D)** *BATF* expression in Tregs in the pleural effusion of HP and LCP patients. **(E)** Differences in Th1 cytokines (IL-12p70, IFN-γ, and TNF-α) between the pleural effusion of HP and LCP. **(F)** Levels of Th2 cytokines (IL-10, IL-4, and IL-5) in the pleural effusion of HP and LCP. **(G)** Levels of inflammatory cytokines (IL-6 and IL-8) in the pleural effusion of HP and LCP. **(H)** Th1/Th2 cytokine ratio comparing TNF-α to IL-10, IL-4, and IL-5 in the pleural effusion of HP and LCP. **(I)** Kaplan-Meier survival analysis of high and low TNF-α/IL-10 ratios. HR: hazard ratio; ns: not significant; * *p* < 0.05; ** *p* < 0.01; **** *p* < 0.001.

Tregs are the most prominent immunosuppressive cells in the tumor microenvironment, and accumulating evidence suggests that their heterogeneity contributes to distinct suppression mechanisms on CD4 T cells 23. Therefore, we analyzed the phenotype of Tregs in the MPE of LCP. Flow cytometry analysis revealed that the number of Tregs was greater in the MPE of LCP than in the pleural effusion of HP (Figure 6A). Since these pleural microenvironment Tregs expressed the Th1 and Th2-regulated transcription factor *TBX21* (Th1) and *GATA2* (Th2*)*, but not* RORC* (Th17), they were inferred to be Th1 and Th2-like Tregs (Figure 6B). These pleural Tregs expressed the exhaustion markers, *CTLA4*, *TIGIT*, and the regulatory transcription factor *BATF*. More importantly, compared to the Tregs in the pleural effusion of HP, the Tregs in the MPE of LCP exhibited higher expression levels of *CTLA4, TIGIT,* and* BATF* (Figures 6C and 6D).

Using the Luminex system to analyze Th1/Th2 cytokines in pleural effusion, we found that Th1 cytokines, including TNF-α, IFN-γ, and IL-12, were significantly lower in the MPE of LCP compared to the pleural effusion of HP (Figure 6E). However, Th2 cytokines, including IL-10, IL-4, and IL-5, did not reach statistical significance (Figure 6F). IL-8, but not IL-6 levels, were significantly elevated in the MPE of LCP (Figure 6G). Various Th1/Th2 ratios, including TNF-α/IL-10, TNF-α/IL-4, TNF-α/IL-5, IFN-γ/IL-4, and IL-12/IL-4, were significantly lower in the MPE of LCP than in the pleural effusion of HP (Figure 6H and sFigure 5A and 5B). This suggests that the metastatic pleural niche may exhibit a Th2-skewed immune environment. Further analysis of individual Th1 and Th2 cytokines revealed that most cytokines were not associated with overall survival rates (sFigure 5C-E). However, a higher TNF-α/IL-10 ratio was associated with better overall survival (HR = 0.53, *p* = 0.1554), although this was not statistically significant (Figure 6I).

### High concentrations of cell-cell interaction factors, BAG6 and IL-7, affect the overall survival rate of lung cancer patients with pleural effusion

**Figure 7:**
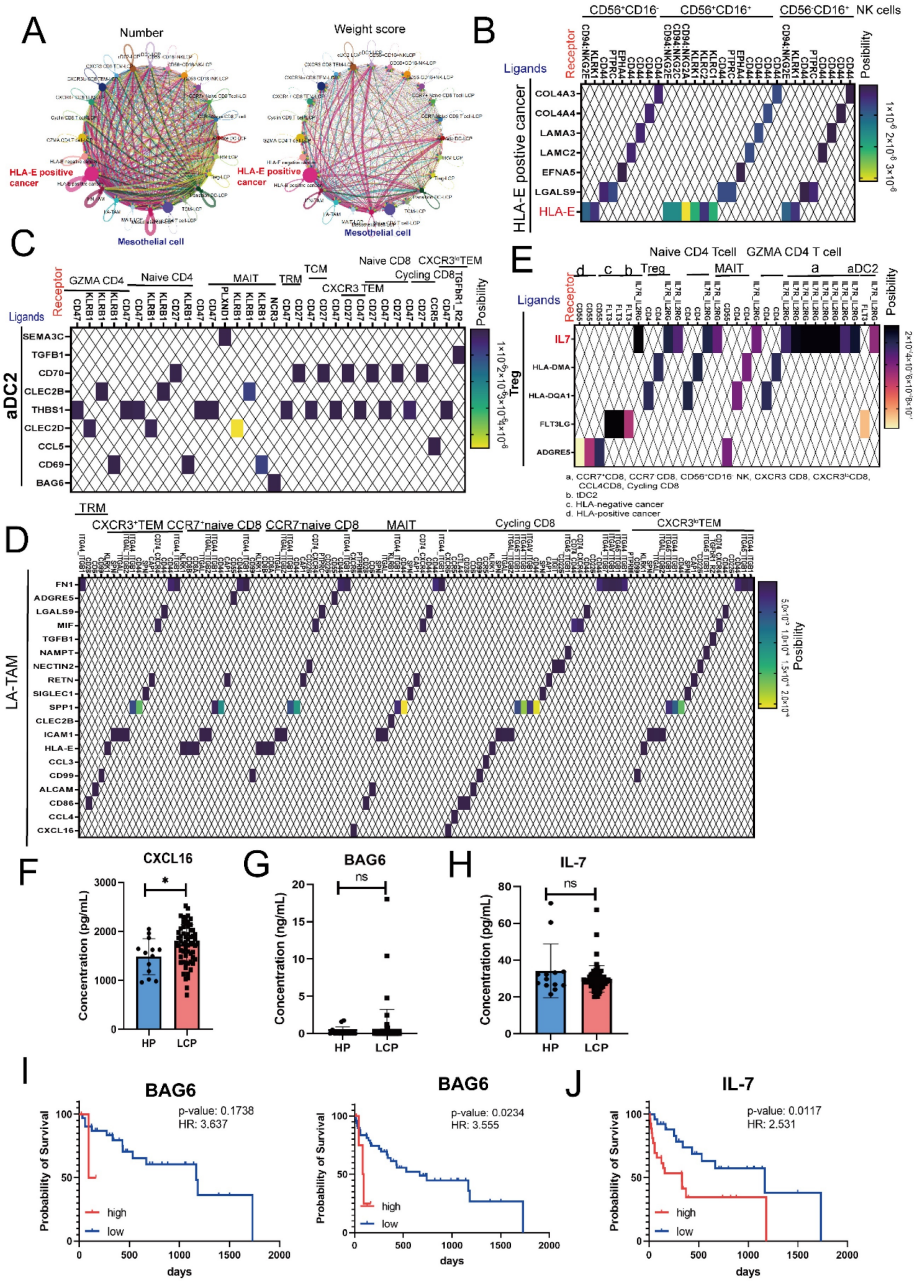
** Cell-cell interactions predict poor survival biomarkers in MPE.** (A) Number and weight scores of interactions between DCs, LA-TAMs, NK cells, T cells, mesothelial and cancer cells. (B) Ligand-receptor interactions between HLA-E positive cancer cells and NK cells. (C) Interactions between aDC2 and various subsets of CD4 and CD8 T cells. (D) Heatmap showing ligand-receptor interactions from LA-TAMs to various subsets of CD8 T cells. (E) Relationship between Tregs and CD4/CD8 clusters as well as cancer cells. Expression levels of CXCL16 (F), BAG6 (G), and IL-7 (H) in the pleural effusion of HP and LCP. Survival analysis of BAG6 (total: right; EGFR mutation: left) (I) and IL-7 (J) in lung cancer patients with MPE. HR: hazard ratio; ns: not significant; *, *p* <0.05.

Finally, we used CellChat to analyze the specific interactions within the MPE microenvironment. As shown in Figure 7A, in terms of both the number and weight score of cell-cell interactions, cancer cells, particularly HLA-E-positive cancer cells, played a crucial role in shaping the pleural microenvironment, while mesothelial cells played a secondary role. Consistent with previous findings, CellChat analysis indicated that HLA-E-positive cancer cells can regulate NK cell function through the binding of inhibitory receptors CD94/NKG2E via HLA-E in all subsets of NK cells (CD56⁺CD16⁻, CD56⁺CD16⁺, and CD56⁻CD16⁺) (Figure 7B). Similarly, cancer cells also influenced all CD8 T cell clusters through the expression of HLA-E (sFigure 6A). Moreover, HLA-E-positive cancer cells interacted with mesothelial cells via various cell-matrix proteins, including collagen and TNC (sFigure 6B). Since previous results showed that APCs, specifically aDC2, exhibited an exhausted phenotype in MPE, we analyzed their cell-cell interactions with CD8 and CD4 T cells. The results revealed that aDC2 influences various CD4 and CD8 T cells through *THBS1*, affects cycling CD8 T cell function via the *CCL5-CCR5* axis, and impacts MAIT cells through *BAG6* (Figure 7C). Another immunosuppressive APC, LA-TAMs, was also found to regulate multiple CD4 and CD8 T cell subsets through immune inhibitory factors, including *SPP1, MIF,* and *LGALS9* (Figure 7D and sFigure 6C). Additionally,* TGF-β* specifically regulated CXCR3^lo^ CD8 TEM cells, while *CXCL16* had a more selective regulatory effect on Cycling, MAIT, and GZMA CD4 T cells (Figure 7D and sFigure 6C). LA-TAMs also influenced NK cells via the same molecules—*SPP1, MIF, LGALS9,* and *TGF-β* (sFigure 6D).

Beyond APCs, CellChat results showed that Tregs influenced multiple lymphoid cell types. Specifically, through IL-7 secretion, Tregs regulated NK cells, CD4 T cells, and CD8 T cells simultaneously and provided feedback to APCs, particularly aDC2 (Figure 7E). More importantly, regardless of CD4, CD8, or aDC2 clusters, the expression of the IL-7 receptor (IL7R) was higher in LCP than in HP (sFigure 6E). Our previous research confirmed that molecules such as *LGALS9, MIF,* and* RETN* were significantly elevated in the MPE of LCP compared to the pleural effusion of HP. Therefore, we conducted a quantitative analysis of other communication factors involved in lymphoid cell regulation, including IL-7, CXCL16, and BAG6. The results showed that CXCL16 was significantly higher in the MPE of LCP than in the pleural effusion of HP, whereas BAG6 and IL-7 did not exhibit statistically significant difference (Figure 7F-H). However, we found that, regardless of EGFR mutation status, high pleural BAG6 levels were associated with poorer overall survival in lung cancer patients with MPE regardless of EGFR status (Figure 7I). Additionally, high IL-7 levels correlated with poor survival rates in the overall patient cohort (Figure 7J). However, in EGFR-mutated patients, despite an HR of 2.182, the association was not statistically significant (sFigure 6F).

## Discussion

**Figure 8:**
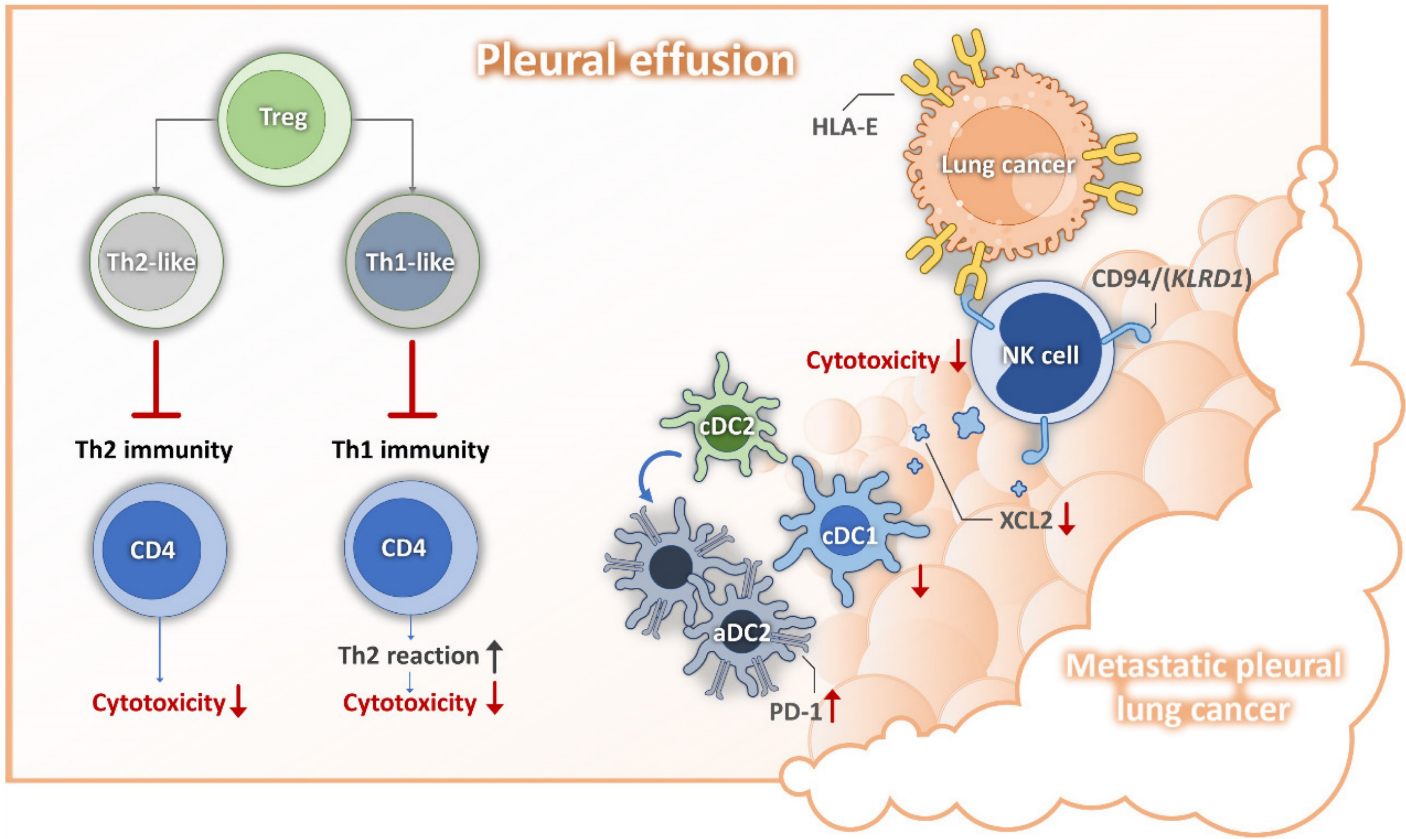
** Mechanisms for lung cancer pleural metastasis.** DC, dendritic cell; HLA-E, human leukocyte antigen; NK, nature killer cell; PD1, programmed cell death protein 1; Th, helper T cell;

Pleural metastasis is associated with poor prognosis and significantly impacts quality of life in patient with lung cancer 24, 25. Our previous research indicated that mesothelial cell mesothelial-to-mesenchymal transition (mesoMT) and the immunosuppressive function of lipid associated-tumor associated macrophages, (LA-TAMs) play crucial roles in shaping the metastatic pleural microenvironment 15. Building upon these findings, our current study focuses on the alterations in lymphoid cell populations within this microenvironment. We identified several key molecular mechanisms, including the role of senescent cancer cell-expressed HLA-E in impairing NK cell cytotoxicity and reducing the recruitment of cDC1, leading to a diminished population of this critical antigen-presenting cell subset. Moreover, we observed that aDC2 exhibited an exhausted phenotype, further contributing to the immunosuppressive milieu. Additionally, the presence of Tregs disrupted the Th1/Th2 balance, fostering an environment unfavorable for anti-tumor immune responses (depicted in Figure 8). Based on these findings, we propose that targeting cancer cell senescence or HLA-E-mediated immunosuppressive events may serve as a promising strategy to restore anti-tumor immunity within the pleural metastatic niche.

It is now widely recognized that various types of cancer cells can still be driven into senescence through different cancer treatments, including chemotherapy, radiotherapy, and targeted therapy 26. Growing evidence suggests that proinflammatory senescence-associated secretory phenotype (SASP) factors released by senescent cells can enhance the proliferation, invasion, and migration of non-senescent cancer cells 27. Additionally, these factors contribute to tumor progression by promoting cancer-supportive angiogenesis and shielding the tumor from immune clearance through immunosuppressive mechanisms 28. Previous studies have confirmed that alterations in major histocompatibility complex (MHC) expression can enable senescent cells to evade immune system recognition, a phenomenon previously observed in cancer and virus-infected cells 29, 30. Pereira *et al.* reported that senescent dermal fibroblasts express the HLA-E, which binds to the inhibitory receptor, NKG2A, found on NK cells and CD8 T cells, thereby suppressing immune responses against senescent cells 31. HLA-E has been proven to participate in the immune evasion mechanism of cancer cells. Circulating tumor cells can enhance HLA-E expression and bind to CD94 (KLRD1)/NKG2A on NK cells, thereby weakening NK-mediated tumor cell killing and cDC1 recruitment via XCL2 secretion. Disrupting the interaction caused by HLA-E can restore the anti-cancer effects of NK cells and prevent cancer metastasis *in vivo* 32. In our study, we found that metastatic pleural cancer cells with high expression of HLA-E exhibited a senescence phenotype. These cells interacted with two major NK cell subsets in MPE—CD56⁺CD16⁺ and CD56⁺CD16⁻—through CD94 (KLRD1), ultimately suppressing the cytotoxicity of these NK cells. Moreover, our results indicated that *HLA-E*-expressing senescent cancer cells have greater immunomodulatory effects on immune cells compared to *HLA-E*-negative cancer cells. This includes various subsets of differentiated effector memory CD8 T cells (TEM), which exhibited reduced cytotoxicity in MPE compared to those found in the pleural effusion of patients with heart failure. Therefore, we propose that targeting HLA-E or employing senolytic therapy may serve as potential therapeutic strategies for treating MPE.

Growing evidence suggests that CD4 T cells can exhibit cytotoxic functions against infected or transformed cells, challenging the conventional belief that CD8 T cells are the primary mediators of tumor cytotoxicity 33, 34. These specialized CD4 T cells can directly kill tumor cells through granzyme B and perforin-dependent mechanisms, FAS-FASL interactions, and TNF-related apoptosis-inducing ligand (TRAIL) pathways 35, 36. Moreover, CD4 T cells with cytotoxicity contribute to the tumor microenvironment by secreting cytokines such as IFN-γ and TNF-α, which enhance immune activation and disrupt immune suppression 37. The cytolytic function of CD4 T cells is associated with Blimp-1 (*PRDM1*) and T-bet (*TBX21*) expression 34, 38 compared to cytotoxic CD4 T cells, which are known to express high levels of granzyme B (*GZMB*), granzyme A (*GZMA*) induces cytolysis in a perforin-dependent, but FAS-FASL-independent manner in graft-versus-host disease 39. Our study identified two groups of GZMA-expressing CD4 T cells: non-proliferative (GZMA CD4 T) and proliferative CD4 (Cycling GZMA CD4 T) cells. Both populations exhibited cytotoxic ability, as indicated by the expression of* GZMA*, *GZMH*, *GZMM*, and *PRF1*, and produced IFN-γ. Furthermore, the gene set of highly expressed GZMA CD4 T cell showed a better overall survival rate in lung cancer patients, further supporting the anti-tumor function of GZMA CD4 T cells. Compared to GZMA CD4 T cells in pleural effusion without tumors, those in MPE of LCP—whether non-proliferative or cycling GZMA CD4 T cells—exhibited suppressed cytotoxic function and reduced GZMA expression. Several transcription factors, including *KLF12*, *ZEB2*, and *FOXP1*, exhibited higher expression in GZMA CD4 T cells within MPE, suggesting their potential regulatory roles in GZMA expression or the cytotoxic function of CD4 T cells. However, given the limited available evidence, further investigation is required to clarify their precise functions.

Tregs, particularly effector Tregs (eTregs) were considered to have the most potent immunosuppressive functions because they can inhibit a wide range of immune cells, including lymphocytes, various types of macrophages, dendritic cells, and B cells in the TME 40, 41. Tregs exhibited heterogeneity by expressing transcription factors typically associated with Th1 (*TBX21*), Th2 (*GATA*,* IRF4*), or Th17 (*STAT3*, *RORC*) cells, leading to the formation of Th1-like, Th2-like, and Th17-like Tregs 42. These specialized Tregs selectively suppressed the corresponding T-effector subgroup 43, 44. In our study, we observed a significant increase in the number of Tregs within MPE of lung cancer patients. Notably, these Tregs exhibited both Th1- and Th2-like effector phenotypes. Analysis of Th1-related cytokines revealed a marked decrease in IFN-γ, TNF-α, and IL-12 levels in MPE of lung cancer patients, whereas Th2-related cytokines, including IL-10, IL-4, and IL-5, did not show a significant increase. Moreover, we found that the Th1/Th2 ratio was markedly reduced in most cases of MPE, suggesting a dysregulated Th1 immune response within the metastatic pleural microenvironment. Interestingly, we also found that Tregs in MPE expressed IL-7 and actively interacted with NK cells, CD8 T cells, and CD4 T cells. Previous studies have demonstrated that IL-7 can enhance the function of these immune cell populations, thereby exerting antitumor effects 45. However, other reports suggested that IL-7 may also promote tumor cell proliferation and inhibit apoptosis, contributing to a pro-tumorigenic effect 46. Although IL-7 levels were comparable between MPE of lung cancer patients and pleural effusions, higher IL-7 concentrations were associated with poorer survival outcomes in lung cancer patients with MPE. These findings highlighted the complex and potentially dual role of IL-7 in pleural metastasis of lung cancer, warranting further investigation to elucidate its precise impact on tumor progression and immune modulation within the pleural microenvironment.

BCL2-associated athanogene 6 (BAG6) plays a multifaceted role in cellular physiology and tumor progression, including protein quality control, immune regulation, and apoptosis 47. BAG6 is involved in the degradation of misfolded proteins and regulates the biogenesis and transport of tail-anchored proteins to maintain protein homeostasis. Additionally, BAG6 plays a crucial role in NK cell-mediated immune surveillance by acting as a ligand for the NKp30 receptor, thereby modulating NK cell cytotoxicity 48. Studies have shown that BAG6 can be secreted in two distinct forms: extracellular vesicle-associated BAG6 (EV-BAG6) and soluble BAG6 (sBAG6), which have opposing effects on tumor immune evasion. EV-BAG6 enhances NK cell activation and cytotoxic responses, whereas sBAG6 suppresses NKp30 expression and diminishes NK cell-mediated tumor cell killing 49. Clinically, elevated serum sBAG6 levels in patients with gastrointestinal stromal tumors are associated with NKp30 downregulation and poor prognosis 50. Our findings indicated that HLA-E-positive cancer cells, mesothelial cells, and aDC2 regulate MAIT cells through the BAG6-NCR3 axis, with high BAG6 concentrations correlating with worse patient prognosis. These results highlight the potential of BAG6 as a predictive biomarker for overall survival in lung cancer patients with pleural metastasis.

## Conclusion

Our study unveils a novel immunosuppressive mechanism in lung cancer with pleural metastasis, driven by the interplay between cancer cell senescence and HLA-E expression. We demonstrate that senescent cancer cells upregulate HLA-E, which directly impairs NK cell cytotoxicity and suppresses cDC1 recruitment, thereby weakening innate and adaptive anti-tumor immunity. BAG6-mediated immune modulation and IL-7's dual effects on tumor progression were also linked to poor survival. This highlights a previously underexplored role of cancer senescence in actively shaping pleural metastatic niches. These findings suggest that targeting HLA-E and senescent cancer cells could restore anti-tumor immunity by correcting lymphoid dysregulation, offering potential therapeutic strategies for lung cancer with pleural metastasis.

## Supplementary Material

Supplementary figures and tables.
