# Supplementary material for: Senescent Tumoral HLA-E Reshapes Microenvironment through Impairing NK Cell-Dendritic Cell-T Cell Network in Malignant Pleural Effusion from Lung Cancer
Source: Int J Biol Sci. 2025 Aug 11;21(12):5240–57. doi: 10.7150/ijbs.116499 (PMC12435339; doi:10.7150/ijbs.116499)
Supplement: Supplementary file 1 — Supplementary figures and tables. [file ijbsv21p5240s1.pdf]

Supplementary Table

sTable 1. The basic information of the patients for scRNA-seq

**Sample**	**Gender**	**Age**	**Diagnosis**	**Group**	**EGFR mutation**
**1**	F	78	HF	HP	N/A
**2**	M	64	HF	HP	N/A
**3**	M	66	HF	HP	N/A
**4**	F	79	HF	HP	N/A
**5**	F	67	MPE	LCP	Del 19
**6**	F	58	MPE	LCP	L858R
**7**	F	77	MPE	LCP	L858R
**8**	M	70	MPE	LCP	Del 19
**9**	M	72	MPE	LCP	T790M, L858R
Abbreviations: HF: Heart failure; F: female; M: male; MPE: Lung adenocarcinoma with malignant pleural effusion; N/A: not applicable.					

sTable 2. The top 100 genes of GZMA CD4 T cells

FeatureID	FeatureName
ENSG00000099204	ABLIM1
ENSG00000198846	TOX
ENSG00000152270	PDE3B
ENSG00000135127	BICDL1
ENSG00000113088	GZMK
ENSG00000168675	LDLRAD4
ENSG00000185989	RASA3
ENSG00000109320	NFKB1
ENSG00000142541	RPL13A
ENSG00000182162	P2RY8
ENSG00000183049	CAMK1D
ENSG00000153814	JAZF1
ENSG00000008988	RPS20
ENSG00000118503	TNFAIP3
ENSG00000114861	FOXP1
ENSG00000151150	ANK3
ENSG00000198821	CD247
ENSG00000171843	MLLT3
ENSG00000107263	RAPGEF1
ENSG00000118922	KLF12
ENSG00000181555	SETD2
ENSG00000108669	CYTH1
ENSG00000010810	FYN
ENSG00000166233	ARIH1
ENSG00000160584	SIK3
ENSG00000083168	KAT6A
ENSG00000152127	MGAT5
ENSG00000164300	SERINC5
ENSG00000112419	PHACTR2
ENSG00000107771	CCSER2
ENSG00000159023	EPB41
ENSG00000245532	NEAT1
ENSG00000166501	PRKCB
ENSG00000155849	ELMO1
ENSG00000027075	PRKCH

ENSG00000154217	PITPNC1
ENSG00000183943	PRKX
ENSG00000136770	DNAJC1
ENSG00000140455	USP3
ENSG00000154310	TNIK
ENSG00000055208	TAB2
ENSG00000228655	AC079793.1
ENSG00000183826	BTBD9
ENSG00000036257	CUL3
ENSG00000054611	TBC1D22A
ENSG00000147604	RPL7
ENSG00000091039	OSBPL8
ENSG00000156110	ADK
ENSG00000135250	SRPK2
ENSG00000074054	CLASP1
ENSG00000169554	ZEB2
ENSG00000198938	MT-CO3
ENSG00000141376	BCAS3
ENSG00000257242	LINC01619
ENSG00000159733	ZFYVE28
ENSG00000186153	WWOX
ENSG00000150347	ARID5B
ENSG00000145996	CDKAL1
ENSG00000156639	ZFAND3
ENSG00000118007	STAG1
ENSG00000123066	MED13L
ENSG00000152061	RABGAP1L
ENSG00000131018	SYNE1
ENSG00000115977	AAK1
ENSG00000034677	RNF19A
ENSG00000100354	TNRC6B
ENSG00000177565	TBL1XR1
ENSG00000071082	RPL31
ENSG00000181722	ZBTB20
ENSG00000150867	PIP4K2A
ENSG00000145730	PAM
ENSG00000139083	ETV6

ENSG00000055609	KMT2C
ENSG00000152520	PAN3
ENSG00000185305	ARL15
ENSG00000149177	PTPRJ
ENSG00000049618	ARID1B
ENSG00000182774	RPS17
ENSG00000127124	HIVEP3
ENSG00000135048	CEMIP2
ENSG00000078304	PPP2R5C
ENSG00000127152	BCL11B
ENSG00000120071	KANSL1
ENSG00000026508	CD44
ENSG00000179715	PCED1B
ENSG00000131558	EXOC4
ENSG00000171310	CHST11
ENSG00000230590	FTX
ENSG00000164483	SAMD3
ENSG00000100906	NFKBIA
ENSG00000107742	SPOCK2
ENSG00000141506	PIK3R5
ENSG00000106415	GLCCI1
ENSG00000111913	RIPOR2
ENSG00000197756	RPL37A
ENSG00000197442	MAP3K5
ENSG00000116539	ASH1L
ENSG00000115464	USP34
ENSG00000092199	HNRNPC

Supplementary Figures

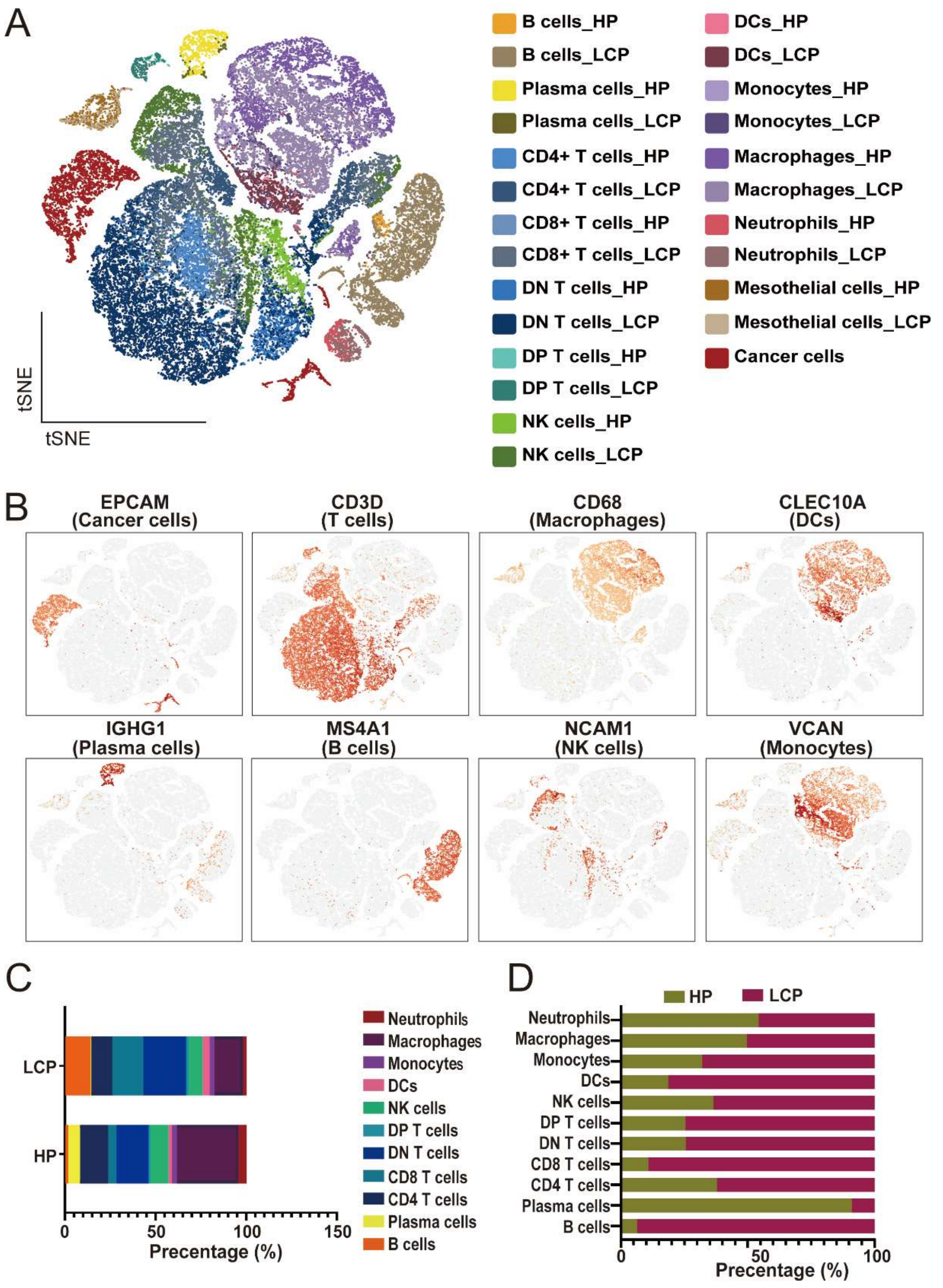

sFigure 1

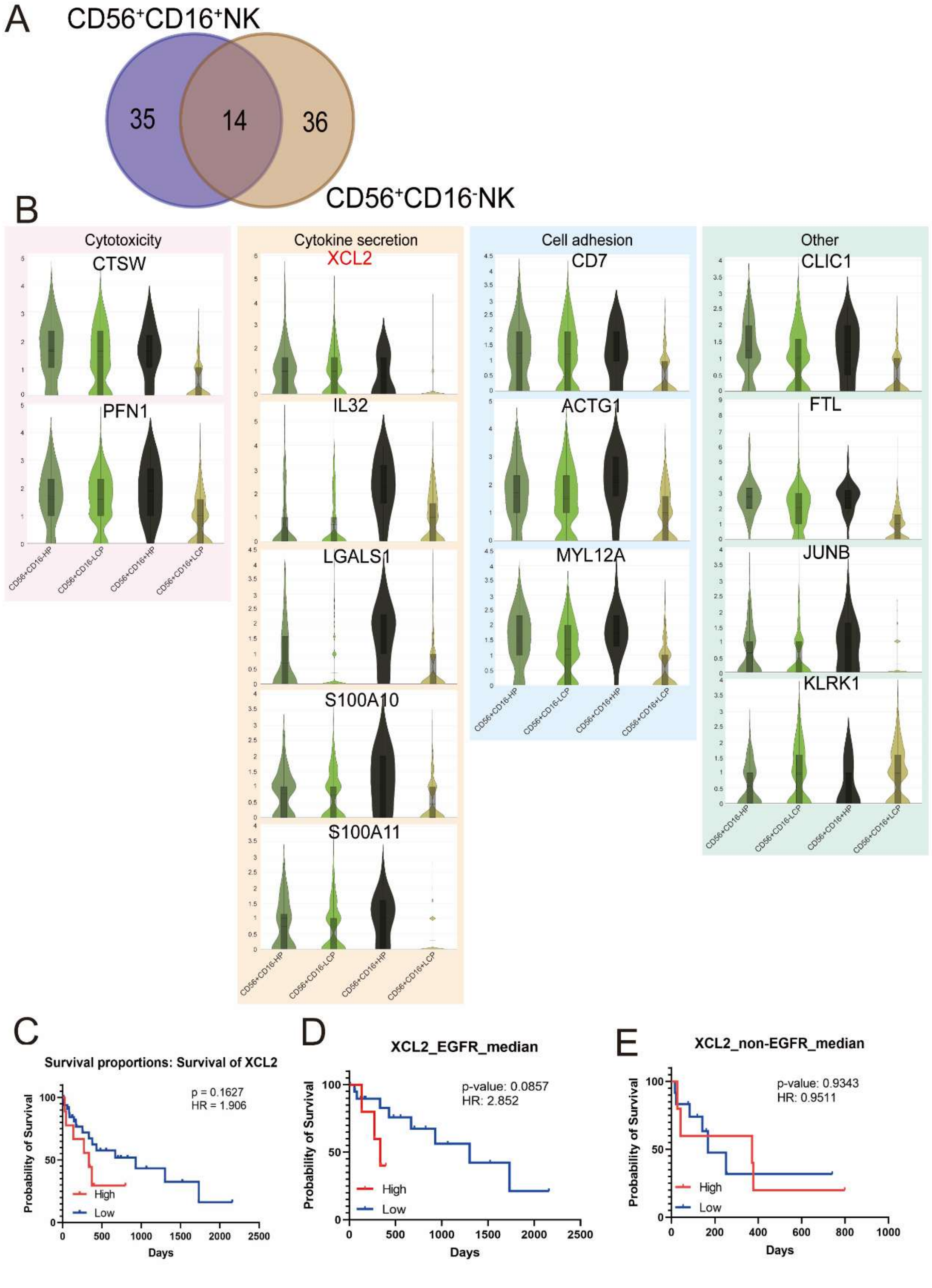
A
CD56+CD16+NK
35
14
36
CD56+CD16-NK
B
Cytotoxicity
CTSW
PFN1
Cytokine secretion
XCL2
IL32
LGALS1
S100A10
S100A11
Cell adhesion
CD7
ACTG1
MYL12A
Other
CLIC1
FTL
JUNB
KLRK1
CD56+CD16-HP
CD56+CD16-LCP
CD56+CD16+HP
CD56+CD16+LCP
C
Survival proportions: Survival of XCL2
p = 0.1627
HR = 1.906
Probability of Survival
Days
High
Low
D
XCL2_EGFR_median
p-value: 0.0857
HR: 2.852
E
XCL2_non-EGFR_median
p-value: 0.9343
HR: 0.9511

sFigure 2

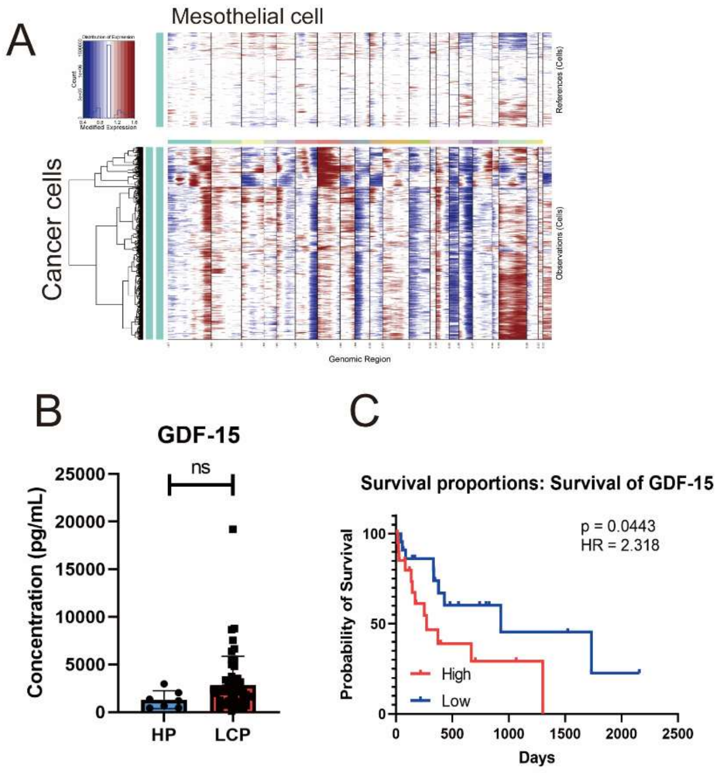
A
Mesothelial cell
Cancer cells
References (Cells)
Observations (Cells)
Genomic Region
B
GDF-15
ns
Concentration (pg/mL)
25000
20000
15000
10000
5000
0
HP
LCP
C
Survival proportions: Survival of GDF-15
p = 0.0443
HR = 2.318
Probability of Survival
100
50
0
High
Low
0
500
1000
1500
2000
2500
Days

sFigure 3

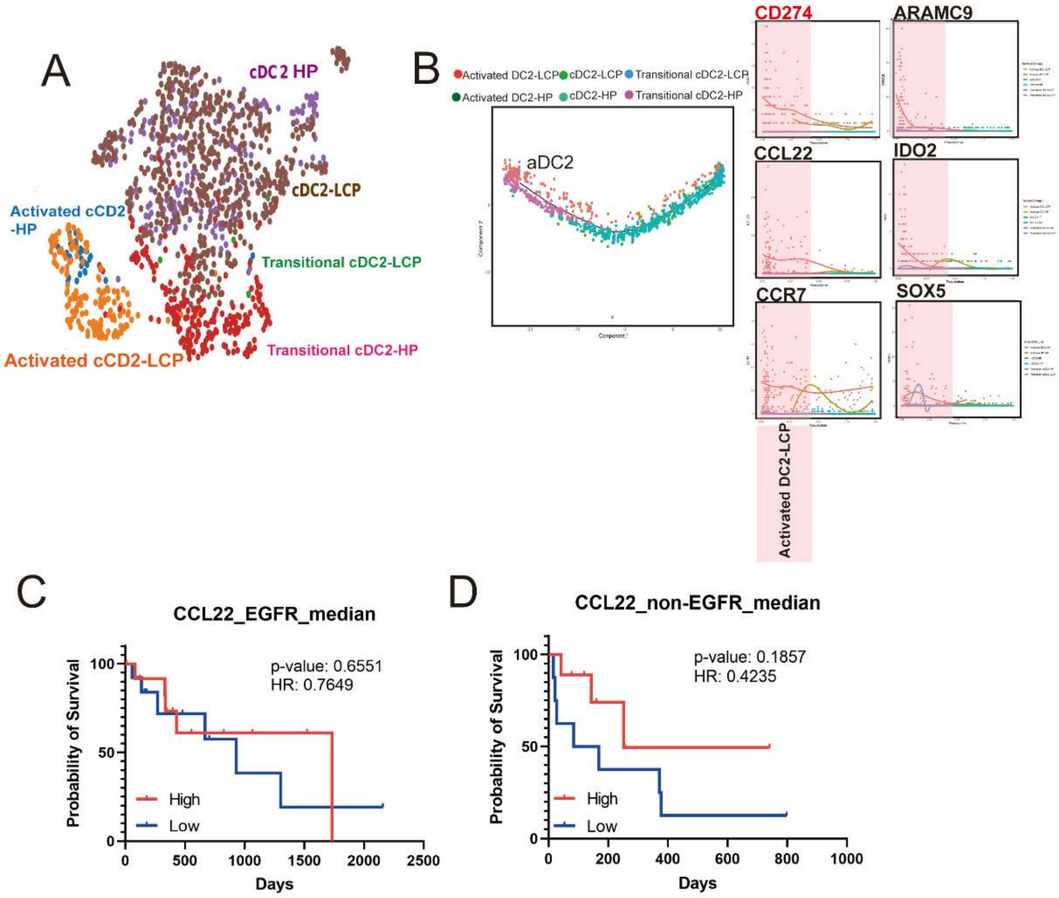

A
cDC2 HP
cDC2-LCP
Activated cCD2
-HP
Transitional cDC2-LCP
Activated cCD2-LCP
Transitional cDC2-HP
B
Activated DC2-LCP
cDC2-LCP
Transitional cDC2-LCP
Activated DC2-HP
cDC2-HP
Transitional cDC2-HP
aDC2
CD274
ARAMC9
CCL22
IDO2
CCR7
SOX5
Activated DC2-LCP
C
CCL22_EGFR_median
p-value: 0.6551
HR: 0.7649
Probability of Survival
High
Low
Days
D
CCL22_non-EGFR_median
p-value: 0.1857
HR: 0.4235
Probability of Survival
High
Low
Days

sFigure 4

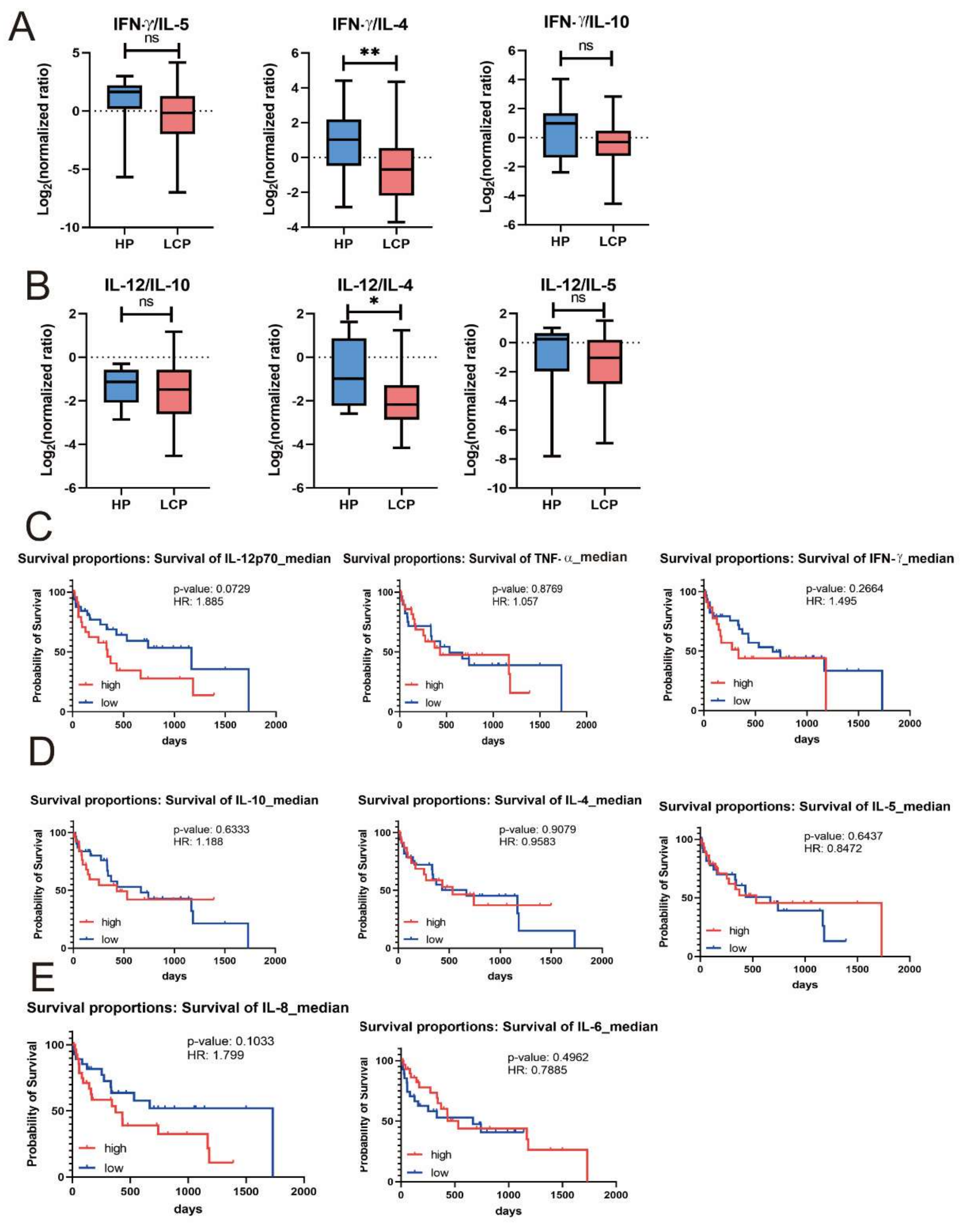
A
IFN-γ/IL-5
IFN-γ/IL-4
IFN-γ/IL-10
Log2(normalized ratio)
ns
**
HP
LCP
B
IL-12/IL-10
IL-12/IL-4
IL-12/IL-5
*
C
Survival proportions: Survival of IL-12p70_median
p-value: 0.0729
HR: 1.885
Survival proportions: Survival of TNF-α_median
p-value: 0.8769
HR: 1.057
Survival proportions: Survival of IFN-γ_median
p-value: 0.2664
HR: 1.495
D
Survival proportions: Survival of IL-10_median
p-value: 0.6333
HR: 1.188
Survival proportions: Survival of IL-4_median
p-value: 0.9079
HR: 0.9583
Survival proportions: Survival of IL-5_median
p-value: 0.6437
HR: 0.8472
E
Survival proportions: Survival of IL-8_median
p-value: 0.1033
HR: 1.799
Survival proportions: Survival of IL-6_median
p-value: 0.4962
HR: 0.7885
Probability of Survival
days
high
low

sFigure 5

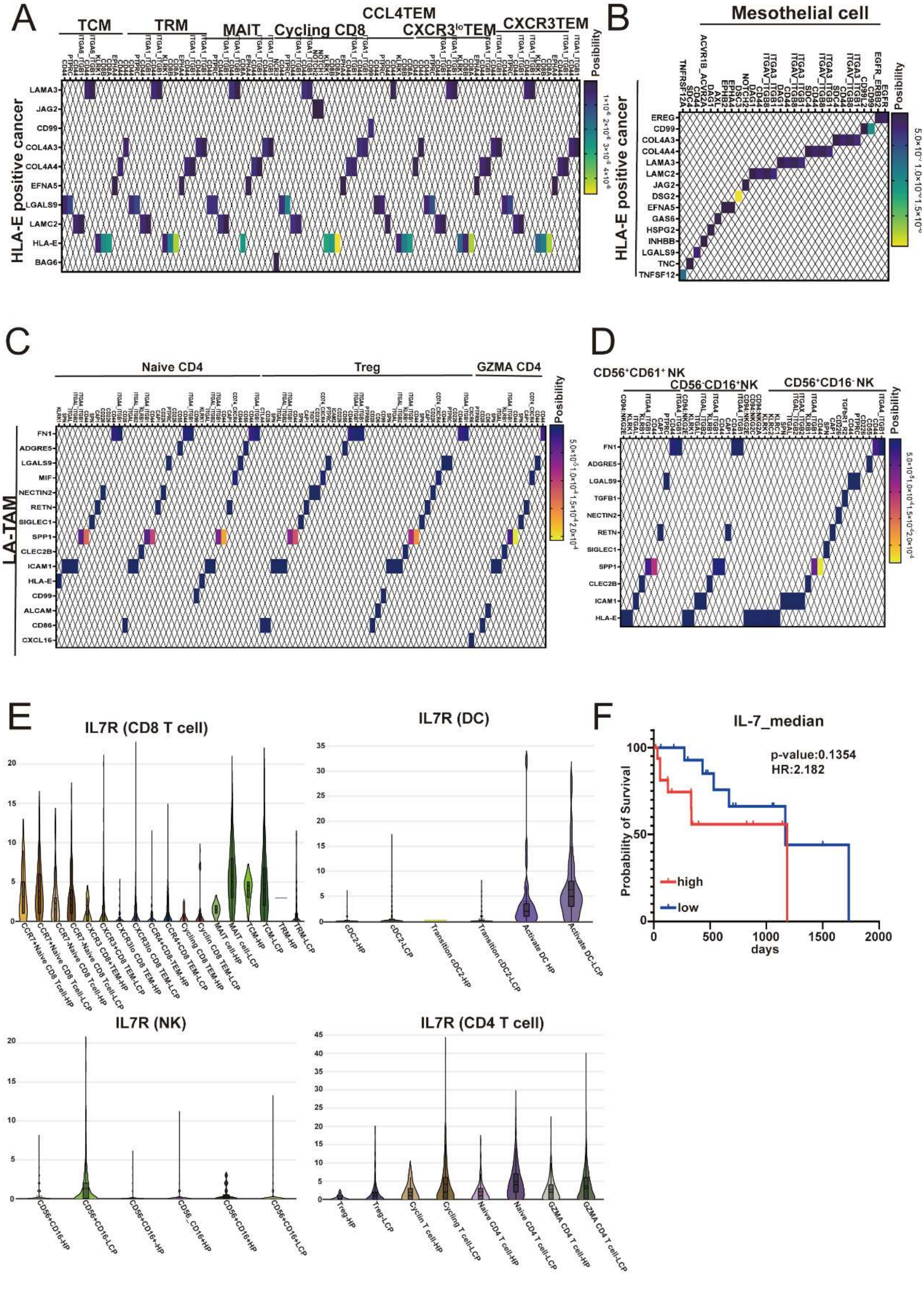
A
TCM
TRM
MAIT
Cycling CD8
CCL4TEM
CXCR3loTEM
CXCR3TEM
HLA-E positive cancer
LAMA3
JAG2
CD99
COL4A3
COL4A4
EFNA5
LGALS9
LAMC2
HLA-E
BAG6
Posibility
B
Mesothelial cell
HLA-E positive cancer
EREG
CD99
COL4A3
COL4A4
LAMA3
LAMC2
JAG2
DSG2
EFNA5
GAS6
HSPG2
INHBB
LGALS9
TNC
TNFSF12
Posibility
C
Naive CD4
Treg
GZMA CD4
LA-TAM
FN1
ADGRE5
LGALS9
MIF
NECTIN2
RETN
SIGLEC1
SPP1
CLEC2B
ICAM1
HLA-E
CD99
ALCAM
CD86
CXCL16
Posibility
D
CD56+CD61+ NK
CD56-CD16+NK
CD56+CD16- NK
FN1
ADGRE5
LGALS9
TGFB1
NECTIN2
RETN
SIGLEC1
SPP1
CLEC2B
ICAM1
HLA-E
Posibility
E
IL7R (CD8 T cell)
IL7R (DC)
IL7R (NK)
IL7R (CD4 T cell)
F
IL-7_median
p-value:0.1354
HR:2.182
Probability of Survival
high
low
days

sFigure 6

Supplementary Figure Legends

Figure S1. Blueprint of major clusters in pleural effusion. (A) Annotation of immune and non-immune cells in pleural effusion of HP and LCP. (B) Heatmap demonstrating cell-specific markers used for cluster classification. (C) Proportional distribution of clusters in in pleural effusion of HP and LCP. (D) Comparison of cell counts in each cluster.

Figure S2. Gene profile of two NK cell subsets. (A) Venn diagram illustrating fourteen commonly low-expressed genes in $CD56^{+}CD16^{+}$ and $CD56^{+}CD16^{-}$ NK cell clusters. (B) Violin plots depicting these fourteen genes based on NK cell functions, including cytotoxicity, cytokine secretion, and cell adhesion. (C) XCL2 levels were not associated with overall survival in lung cancer patients with malignant pleural effusion (MPE) ($p = 0.1627$). (D) No association was observed between XCL2 levels and overall survival in patients with EGFR mutations ($p = 0.0857$). (E) Similarly, no association was found in patients with EGFR wild-type ($p = 0.9511$).

Figure S3. The levels of GDF-15. (A) The InferCNV of pleural metastatic cancer. (B) The level of GDF-15 in pleural effusion of HP and LCP by ELISA. (C) Higher GDF-15 was associated with worse OS in lung cancer patient with MPE ($p = 0.0443$). (ns, not significant)

Figure S4. Differentiation of cDC2 clusters. (A) tSNE plot identifying three types of cDC2s in the pleural effusion of HP and LCP. (B) Trajectory and pseudotime analysis showing six upregulated genes contributing to the transition of cDC2 subsets. (C) Kaplan–Meier analysis revealed that CCL22 levels in MPE were not significantly associated with overall survival in lung cancer patients with EGFR mutation (C) (HR = 0.7649, $p = 0.6551$) and wild-type patients (D) (HR = 0.4235, $p = 0.1857$).

Figure S5. Comparison of Th1/Th2 ratios and survival analysis via ELISA. (A) Ratio of IFN-γ to IL-5, IL-4, and IL-10 in pleural effusion of HP and LCP. (B) Ratio of IL-12 to IL-10, IL-4, and IL-5 in pleural effusion of HP and LCP. Kaplan-Meier survival analysis of Th1 (C), Th2 (D), and inflammatory cytokines (E) in lung cancer patient with MPE. (ns: not significant; *, <0.05; **, <0.01)

Figure S6. Interaction of *HLA-E* positive cancer cells and LA-TAMs with other clusters. (A) Interaction of *HLA-E* positive cancer cells with T cell subsets. (B) Cell-cell interactions between *HLA-E* positive cancer cells and mesothelial cells. (C) Ligand-receptor interactions from LA-TAMs to CD4 T cells. (D) Ligand-receptor interactions from LA-TAMs to NK cells. (E) Violin plots illustrating IL-7R expression across CD4/CD8 T cells, DC, NK and cancer cells. (F) Kaplan-Meier survival analysis of IL-7 median expression and its correlation with overall survival in EGFR-mutated patients.
